# Robotic Centrifugal Microfluidics with In-Rotation Liquid Supply for the Extraction of Multiple Liquid Biopsy Analytes in One Platform

**DOI:** 10.3390/bios16060309

**Published:** 2026-05-28

**Authors:** Truong-Tu Truong, Yumi Kaku, Gonzalo Bustos-Quevedo, Sara ElGenk, Ehsan Mahmodi Arjmand, Gustav Grether, Jan Lüddecke, Judith Schlanderer, Stefan Wagner, Theresa Katschmareck, Eva Dazert, Nikolas von Bubnoff, Irina Nazarenko, Germán Matías Hansen, Sabrina Kartmann, Tobias Hutzenlaub, Nils Paust, Peter Juelg

**Affiliations:** 1 Hahn-Schickard, 79110 Freiburg, Germanyehsan.arjmand@hahn-schickard.de (E.M.A.); gustav.grether@hahn-schickard.de (G.G.); jan.lueddecke@hahn-schickard.de (J.L.); judith.schlanderer@hahn-schickard.de (J.S.); irina.nazarenko@uniklinik-freiburg.de (I.N.); matias.hansen@hahn-schickard.de (G.M.H.); sabrina.kartmann@hahn-schickard.de (S.K.); tobias.hutzenlaub@hahn-schickard.de (T.H.); nils.paust@hahn-schickard.de (N.P.); peter.juelg@hahn-schickard.de (P.J.); 2 Institute for Infection Prevention and Control, Medical Center, Faculty of Medicine, 79106 Freiburg, Germany; gonzalo.salvador.bustos.quevedo@uniklinik-freiburg.de; 3 Faculty of Biology, University of Freiburg, 79104 Freiburg, Germany; 4 Hahn-Schickard, 70569 Stuttgart, Germany; stefan.wagner@hahn-schickard.de; 5 Department of Hematology and Oncology, University Medical Center Schleswig-Holstein, Campus Lübeck, 23538 Lübeck, Germany; theresalilith.katschmareck2@uksh.de (T.K.); eva.dazert-klebsattel@uksh.de (E.D.); nikolaschristiancornelius.vonbubnoff@uksh.de (N.v.B.); 6 University Cancer Center Schleswig-Holstein, University Medical Center Schleswig-Holstein, Campus Lübeck, 23538 Lübeck, Germany; 7 European Liquid Biopsy Society (ELBS), 20246 Hamburg, Germany; 8 Laboratory for MEMS Applications, IMTEK—Department of Microsystems Engineering, University of Freiburg, 79110 Freiburg, Germany

**Keywords:** centrifugal microfluidics, robotic, lab-on-chip, cell-free DNA (cfDNA), extracellular vesicles, instrumentation, automation, liquid handling, liquid biopsy, valving, multi-analyte, sample preparation

## Abstract

**Background**: The growing demand for versatile laboratory automation is exemplified in the context of liquid biopsy, where multi-analyte approaches are increasingly recognised for their potential to enhance diagnostic sensitivity in oncology. However, current practice often necessitates the use of dedicated instruments and workflows for the extraction of each analyte, posing financial and logistical barriers for automated multi-analyte liquid biopsy. **Methods**: Here, we present Robotic Centrifugal Microfluidics (*RoCM*), an all-in-one platform that combines the versatility of centrifugal microfluidics and operational flexibility of robotic liquid handling. This combination enables the automation of complex micro- and macrofluidic protocols, realised through the use of (1). exchangeable microfluidic cartridges and (2). programmable robotic operations such as in-rotation liquid supply, magnetic bead manipulation, or microfluidic valving. In-rotation robotic liquid manipulation maintains fluid control under centrifugal forces and reduces the cartridge footprint associated with pre-loaded liquid reservoirs. Platform applicability was demonstrated using two exemplary liquid biopsy workflows: the extraction of cell-free DNA (cfDNA) from blood plasma using *RoCM-cfDNA slices* and the extraction of extracellular vesicles (EVs) from blood plasma using *RoCM-EV slices*. **Results**: In a pilot study with patient samples from different cancer entities, the *RoCM-cfDNA slices* yielded comparable variant allele frequencies to a commercial bead-based instrument, while the *RoCM-EV slices* achieved a recovery of a greater diversity of EV subpopulations than semi-automated size-exclusion chromatography. **Conclusions**: By simply exchanging cartridges, *RoCM* enables the extraction of diverse analytes within a single automated system. Its application can be extended to further analytes, such as circulating tumour cells (CTCs), or to applications beyond liquid biopsies, where versatile micro- and macrofluidic protocols benefit from implementation in a single automation instrument.

## 1. Introduction

Extensive research has been conducted to leverage blood-based analytes for the detection and monitoring of cancer, a minimally invasive approach termed liquid biopsy [1]. Among these analytes, the most extensively studied are circulating tumour cells (CTCs) [2,3,4,5] that are shed by tumours as intact cells, lipid-bilayer enclosed particles containing nucleic acids, proteins, and lipids, called extracellular vesicles (EVs) [6,7,8,9], and cell-free DNA (cfDNA) [10,11,12,13,14], which constitutes fragmented nucleosomal DNA. All these different analytes carry molecular information that can reflect tumour dynamics and support therapeutic decision-making [1].

While early efforts predominantly targeted individual analytes, increasing evidence suggests that multi-analyte information may be essential to fully realise the clinical potential of liquid biopsy [1]. This broadened perspective is founded by a growing body of literature demonstrating improved diagnostic performance through the integration of multiple parameters compared to single-analyte approaches [15,16,17].

Among the available automation solutions for blood-based analyte extraction, two promising principles stand out: robotic liquid handling workstations and microfluidic chips, each presenting unique advantages and limitations in the context of multi-analyte liquid biopsy applications.

Robotic liquid handling workstations offer high sample parallelisation, a high degree of programmability, and the capability to process large sample volumes, a prerequisite in liquid biopsy applications due to the diluted nature of the tumour-derived analytes in circulation. As an example, for cfDNA extraction, these systems have been widely adopted for silica bead-based bind–wash–elute protocols and are commercially available [18,19]. Lehle et al. [19] demonstrated, in a comparative study on automated cfDNA extraction, that the EZ2 Connect instrument (QIAGEN) yielded the highest cfDNA recovery, offering fully automated purification of up to 24 samples in parallel from sample volumes up to 8 mL. However, this platform is limited to nucleic acid extraction protocols.

The extraction of plasma-derived EVs is inherently more complex, often requiring orthogonal separation methods based on particle size, density, charge or surface molecules [20,21]. Currently, semi-automated solutions are limited to a single separation method, e.g., size-exclusion chromatography (SEC)—using the automatic fraction collector (IZON)—or density gradient-based EV extraction using the Biomek 4000 (Beckman Coulter), as shown by Dorpe and colleagues [22]. The latter has been used with a custom-made script to automate column preparation and fraction collection. The study emphasises the reduced variability by using the automated system compared to manual processing. However, key steps such as centrifugation and SEC are not integrated into current liquid handling systems.

Microfluidic platforms have been increasingly applied to liquid biopsy applications. These platforms encompass a diverse set of mechanisms, including digital microfluidics (DMF) based on electrowetting, electrokinetic platforms leveraging dielectrophoresis (DEP), and centrifugal microfluidics [23]. Droplet-based systems, such as the DMF platform reported by Tong et al. [24] demonstrate automated magnetic bead-based capture of EVs. Yet, their utility is constrained by the limited plasma volume of 20–40 μL and the inherent dependence on affinity-based isolation, which may not comprehensively capture heterogeneous EV subpopulations. Electrokinetic approaches, particularly DEP, have been explored for CTC enrichment, but mostly miss bulk-sample processing steps such as centrifugation, as reviewed by Gascoyne and colleagues [25]. Ibsen et al. introduced a versatile electrokinetic platform [26], subsequently demonstrating the isolation of both cfDNA [27] and EVs [28]. While promising, this approach remains fundamentally restricted to analyte separation based on dielectric properties, and does not yet support orthogonal modalities such as size-based filtration or affinity-driven extraction, thereby limiting its use for broader multi-analyte workflows.

Centrifugal microfluidics presents a promising automation principle for liquid biopsy protocols. It offers a high degree of flexibility in terms of unit operations, including filtration and centrifugation capabilities, which are essential for density- and size-based separation methods. A recent study from our group proposed a centrifugal microfluidic cartridge for cfDNA extraction from 1 mL of blood plasma, integrating pre-stored buffers and thermopneumatic-actuated microfluidic valves [29]. However, the large reagent volumes take up substantial cartridge area, allowing for the processing of only one sample at a time. Kim et al. [30] devised their centrifugal microfluidic cartridge to recover cells with a size larger than 8 µm, including CTCs from 3 mL of blood using sequential filtration. Sunkara et al. [31] demonstrated, on the same platform, its utility for EV extraction using sequential filtration, effectively isolating vesicles between 100 nm and 600 nm from 30–600 µL of blood. On the same platform, cfDNA extraction has been demonstrated from 3 mL of blood [32]. While this system has demonstrated great flexibility in analyte extraction, manual pipetting is still required for sample and buffer loading, and the limitation of processing one patient sample at a time remains.

Given the trade-offs associated with existing liquid handling robots as well as stand-alone microfluidic automation platforms, there remains an unmet need for a system that (1). supports the processing of large sample volumes, (2). enables the processing of more than one sample at a time, and (3). provides orthogonal extraction mechanisms as required for automated multi-analyte liquid biopsy applications in a single instrument. Existing hybrid approaches combining robotic handling with microfluidic modalities, including droplet microfluidics [33,34] or acoustofluidics [35], likewise do not sufficiently address the aforementioned requirements.

In this study, we introduce a novel automation platform, termed Robotic Centrifugal Microfluidics (*RoCM*), combining advantages of robotic liquid handling and centrifugal microfluidic cartridges. *RoCM* is enabled by a new technical solution for the supply of reagents under rotation and two new centrifugal microfluidic valving mechanisms. It is designed to address these limitations by enabling (1). the processing of large sample volumes and (2). a reduced cartridge footprint, as on-demand liquid supply removes the requirement for on-cartridge buffer storage. Most importantly, *RoCM* further enhances (3). flexibility through exchangeable extraction cartridges and exchangeable tools for the robotic arm.

To demonstrate the platform’s applicability for the extraction of multiple analytes, we developed and fabricated two initial *RoCM* cartridge types, one for the extraction of cfDNA and one for EV from plasma, termed *RoCM-cfDNA slice* and *RoCM-EV slice*, respectively. The *RoCM* instrument and both cartridge types were demonstrated on clinical plasma specimens from cancer patients and healthy controls.

## 2. Materials and Methods

### 2.1. Instruments

The platform (Supplementary Figure S1) integrates a 4-axis SCARA robot (i4-450L, Omron), chosen for its high payload capacity for its size class of 5 kg, selected based on a structured decision matrix comparing SCARA and Cartesian (XYZ) architectures (Supplementary Tables S1 and S2). The integrated controller within the robot’s base ensures efficient space utilisation.

A custom-made cartridge rotor (DIALUNOX, Stockach, Germany) was employed to secure the cartridges in place during rotation using vacuum zones located on its upper surface. The rotor also features six independent heating zones, allowing the implementation of various temperature protocols while the system is under rotation.

The rotation module comprises a servo motor (4221X024BXTH IEF3-256 42GPT 14:1 KS7, Faulhaber, Schönaich, Germany) equipped with a 14:1 transmission gearbox and an incremental encoder with 256 counts, controlled via a motor driver (MC5005 S CO, Faulhaber). To enable tool interchangeability during operation, the system utilises a mechanical tool changer (Auto Connector Kit Pneumatic & Electric, GRIP, Dortmund, Germany), which also features electrical contacts to power tools when needed. Continuous power transmission under rotation is facilitated by a 6-wire high-speed slip ring (G2056-06, Senring, Shenzhen, Germany), rated for a maximum rotational speed of 1500 rpm. For calibration of the zero position, a Hall sensor (KY-024, eMagTech, Xiamen, China) in combination with a 2 × 2 × 2 mm Neodym N45 cube magnet (Webcraft GmbH, Gottmadingen, Germany) is used.

The robotic platform supports multiple exchangeable tools. For pipetting applications, Air-Z Flex 1000 µL Horizontal electronic pipettes (TriContinent Scientific, Grass Valley, CA, USA) are integrated, with one pipette positioned at a 10 mm radius from the centre of rotation and the other at 25 mm. Both pipettes are balanced to ensure safe operation under rotation. For magnetic separation applications, a 3D-printed magnet adapter holds five Neodym magnets (15 mm diameter, 8 mm height, 6.2 kg holding force, supermagnete.de) in an equidistant arrangement.

A graphical user interface is displayed on a tablet (Tab M9 TB310FU ZAC30123SE, Lenovo, Beijing, China). The platform is controlled by a Raspberry Pi 5 B (4 × 2.4 GHz, 4 GB RAM, WLAN/BT) running Raspberry Pi OS and programmed in Python 3.7.6 to coordinate all active components. Additionally, a microcontroller (STM32F446RET6, STMicroelectronics, Geneva, Switzerland) supervises the encoder signals from both the tool rotor and the cartridge rotor to ensure precise synchronisation.

### 2.2. Fabrication

**Microfluidic cartridge design and simulation**. The computer-aided design (CAD) model of the cartridges was created using Solidworks Premium 2025 SP3.0 (Dassault Systèmes, Vélizy-Villacoublay, France) for injection moulding production. The performance of the valves was evaluated by modelling microfluidic structures as hydrodynamic networks composed of discrete elements. Network simulations were performed using MATLAB Simulink Simscape R2016a (MathWorks), as previously described [36]. Channel dimensions can be found in Supplementary Figure S2.

**Fabrication of cartridge prototypes**. For the evaluation of fluidics, as well as the characterisation of valve performance and the reproducibility of pipetting under rotation, cartridge prototypes were fabricated from PMMA and COC foils, as previously described [29].

**Fabrication of injection moulded cartridges**. The cartridges were injection moulded with 1 mm thickness from COC (APL6013T, Mitsui Chemicals, Tokyo, Japan). The fluidic layout was precision milled in hardened steel for injection moulding (Allrounder 370A, Arburg, Loßburg, Germany).

**Functionalisation of cartridges**. The incubation chamber of the *RoCM-cfDNA slice* was coated with 200 µL of a 0.5% *v*/*v* Teflon solution (amorphous fluoropolymer, DuPont) dissolved in Fluorinert 770 (Sigma-Aldrich, St. Louis, MO, USA). For the *RoCM-EV slice*, filters were manually integrated by hot embossing with a brass embossing tool. The first filter (19 mm TEPC, 800 nm, cytiva, Marlborough, MA, USA) was embossed using 180 °C, 4.5 bar for 10.4 s. For the second filter, a 0.6 mm thick, fine-grade porous PE support (19 mm diameter, SPC Technologies Ltd., London, UK) was placed beneath a 25 mm AAO 20 nm filter (cytiva, Marlborough, MA, USA), which was embossed using 180 °C, 5 bar, for 10 s. To prevent damage, a Kapton 300HN layer (CMC Klebetechnik GmbH, Frankenthal, Germany) was placed between the embossing tool and the filter during embossing. During sealing, the contact temperature locally exceeded the melting point of COC, resulting in firm fusion of the filter to the surrounding polymer and forming a leak-tight boundary. Leakage testing using fluorescent particle suspensions confirmed effective sealing, and consistent flow rates across cartridges further verified the reproducibility of the integration process; 0.5 mL DNA and Protein LoBind tubes (Eppendorf, Hamburg, Germany) were press-fitted onto the cartridge-tube interfaces of the *RoCM-cfDNA* and *RoCM-EV slices*, respectively.

**Thermal sealing of the injection moulded cartridges.** Thermal sealing of the cartridge was performed using a custom-laminated multi-layer foil (Tekniplex, Wayne, PA, USA) consisting of a COC 8007 sealing layer, a polyurethane middle layer, and a BoPET upper layer, with a total thickness of 72 µm. Sealing was carried out at 170 °C for 12 s, with a sealing force of 3000 N for the *RoCM-EV slice* and 4000 N for the *RoCM-cfDNA slice*.

### 2.3. Evaluation of Robotic Liquid Supply Under Rotation

The evaluation was conducted using a PMMA cartridge with milled measurement chambers designed for 100, 500, and 1000 µL volumes (Supplementary Figure S3). Due to observed volume offsets, the required adjustments were added to the target volumes. The cartridge was sealed with a pressure-sensitive adhesive foil (9795 R, 3 M) featuring laser-cut inlets and air vents. Fluid volumes were dispensed under rotation and centrifuged into the chambers at 20 Hz after each dispensing step. Once dispensing was complete, a PMMA lid was applied to prevent buckling during measurements using a stroboscopic setup (Biofluidix, Freiburg, Germany) at 50 Hz. For comparison, manual pipetting was performed under identical conditions. The transferred volume was evaluated by aligning the stroboscopic images of measurement chambers with the CAD model.

### 2.4. Evaluation of cfDNA Extraction

The protocol details and magnetic bead handling are adapted from Schlenker et al. [29] and outlined in Supplementary Table S1. The performance of the extraction workflows was assessed using qPCR and dPCR. All oligonucleotides were custom-synthesised by biomers.net. Primer and probe sequences used for the detection of wild-type and mutant alleles were previously published by our group [13,37]. Final concentrations of all components as well as thermal cycling conditions are provided in Supplementary Table S2. To more accurately evaluate extraction efficiency, particularly for protein-associated DNA, we used nucleosomes derived from HCT116 cell lines (ctDNA_surrogate_), as DNA in biological samples is typically bound to proteins. The HCT116 cell line was provided by S. Derer (Institute of Nutritional Medicine, University Medical Center Schleswig-Holstein, Campus Lübeck), and nucleosomes were prepared in-house using the Nucleosome Preparation Kit (Active Motif, Carlsbad, CA, USA). The manufacturer’s protocol was followed for 15 million cells, with an optimised 1 h lysis and 15 min enzymatic shearing incubation to digest linker DNA between nucleosomes. All ctDNA_surrogates_ were stored at −80 °C until further use.

### 2.5. Evaluation of EV Extraction

**RoCM-EV-based extraction.** The *RoCM-EV slice* was designed for processing of 400 µL of blood plasma, which is first mixed with 600 µL of PBS-washed CEX Fractogel EMD SO_3_^−^; resin (40–90 µm diameter, Merck KGaA, Darmstadt, Germany) and incubated under rotation for 5 min. This step binds positively charged lipoproteins. The solution is then diluted with 1 mL of sterile filtered PBS before being pipetted onto the cartridge in two 1 mL steps (Supplementary Table S3). Filtration occurs at 25 Hz, with shake-mode mixing every 5 min (10× 5–20 Hz at 24 Hz/s). The samples pass through two filters: first, a TEPC 800 nm filter (cytiva), which removes Fractogel resin, protein aggregates, and large particles, and second, an AAO 20 nm filter (cytiva), which retains EVs. For washing, 1 mL of PBS with 5% SmartBlock solution (Candor Bioscience GmbH, Wangen, Germany) is pipetted in two 500 µL steps, filtered at 25 Hz, with shake-mode mixing. The waste channel is positioned to discard volumes exceeding 100 µL (filtrate), ensuring that 100 µL of the EV concentrate (retentate) is transferred to the protein LoBind 0.5 mL tube (Eppendorf, Hamburg, Germany).

**Recovery experiments.** To characterise the EV recovery from the cartridge, we performed a similar experiment which was published before using CD9-GFP positive EVs [38]. Briefly, EVs were isolated by sequential filtration followed by SEC from an HT1080 transfected cell line expressing CD9-GFP. After isolation, EVs were quantified by F-NTA (QUATT NTA, Particle Metrix GmbH, Meerbusch, Germany) and stored at −80 °C until further use. NTA settings were sensitivity 80, shutter 90 for scatter mode, and sensitivity 95 and shutter 50 for fluorescent mode (software Zetanavigator 1.4.2.1). Samples were diluted in PBS to have between 60 and 200 particles per frame.

**SEC-based extraction**. SEC (qEV original 35 nm gen2, IZON, Christchurch, New Zealand) was used as reference method for EV extraction, following the manufacturer’s instructions. Prior to sample loading, the column was equilibrated with 50 mL of buffer to ensure consistent elution profiles and neutral pH. Subsequently, ten fractions, each at 400 µL, were collected, of which the first two, containing the highest EV concentration based on elution profile, were pooled and compared to the *RoCM-EV* retentate. Samples were stored at −80 °C until further use.

**Evaluation of soluble proteins in EV enriched samples.** To compare the efficiency of soluble protein removal between *RoCM-EV slice* and SEC-based extraction, total protein content was quantified using the Micro BCA Protein Assay Kit (Thermo Scientific, Waltham, MA, USA), following the manufacturer’s instructions. Fresh standards were prepared for each assay, and samples were diluted appropriately to ensure absorbance values within the linear range (0.1–2.0). *RoCM-EV* samples were diluted 1:50, whereas SEC fractions were diluted between 1:10 and 1:50. For SEC, EV-enriched fractions (fractions 1 and 2) and protein-rich fractions (fractions 9 and 10) were identified based on elution profile and subsequently pooled for comparative analysis with the cartridge.

**Evaluation of biomarker expression on EV surface and ApoB on healthy control samples.** To assess the expression of EV surface markers and ApoB in healthy donor samples, enriched EV fractions were stained with fluorophore-conjugated antibodies and analysed using the NanoAnalyzer (NanoFCM, Xiamen, China). The following antibodies were employed: CD61-AF647 (FAB2266R, R&D Systems, Minneapolis, MN, USA) to detect platelet-derived EVs, apoB-FITC (ab27637, Abcam, Cambridge, UK) to evaluate co-isolated lipoproteins, and universal EV markers CD9-APC (312108, BioLegend, San Diego, CA, USA), CD9-FITC (987206, BioLegend) and CD63-FITC (353006, BioLegend).

Instrument calibration was conducted according to the manufacturer’s instructions. Alignment of the laser and concentration calibration were performed using proprietary QC control beads (NanoFCM), and size calibration was achieved using silica nanospheres (68–155 nm, NanoFCM). Dulbecco’s phosphate-buffered saline (Gibco 1× DPBS, Thermo Fisher Scientific, Waltham, MA, USA) was used as the diluent and blank control.

Sample preparation involved incubating 5 µL of isolated EV fractions with 3 µL of antibody (pre-diluted to 6 µg/mL) for 1 h at 4 °C. Following incubation, samples were diluted in 1× DPBS to yield 4000–8000 detectable events per measurement. Acquisition settings were as follows: laser power of 10 mW at 488 nm and 20 mW at 640 nm, sampling pressure at 1 kPa, acquisition time of 1 min, and side scatter (SS) decay set to 10%. Data were analysed using NanoFCM Software (NF profession V2.0) to determine EV concentrations and the proportion of marker-positive events.

**Evaluation of biomarker expression on EV surface from healthy vs patient samples.** To assess the expression of surface biomarkers on EVs, the same healthy donor samples previously analysed by NanoFCM were utilised alongside three additional prostate cancer samples. This approach allowed comparison of the cartridge-based enrichment performance between healthy and cancer-derived samples, with SEC serving as a reference. Multiplexed analysis was performed using the MSD platform (Meso Scale Discovery, Rockville, MD, USA) to quantify five biomarkers: CD14 (monocyte marker), EpCAM (epithelial cell adhesion molecule), and the universal EV markers CD9, CD63, and CD81. The assay was conducted according to the manufacturer’s instructions, using undiluted EV preparations.

**Data analysis and visualization**

Data analysis and visualization of NanoFCM-derived size distributions were performed using the open-source PhoNUPS software (release version) [39].

### 2.6. Clinical Sample Collection

For the spiked-in study, pooled citrate-anticoagulated plasma from healthy donors was collected at the University of Freiburg. Patient samples were collected at the University of Freiburg as citrate-anticoagulated plasma for the EV study and the University Medical Center Schleswig Holstein, Campus Lübeck as EDTA-anticoagulated plasma (Streck tubes, La Vista, NE, USA) for the cfDNA study. Established standard operating procedures were followed to maintain consistent biospecimen quality for this study. All samples were stored at −80 °C until use.

## 3. Results

### 3.1. Concept

#### 3.1.1. RoCM Platform Architecture

**Figure 1:**
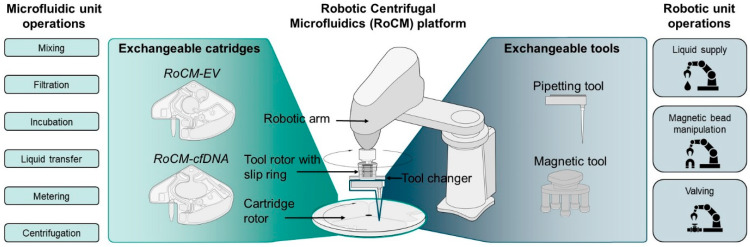
**RoCM platform architecture.** Modularity is realised through exchangeable cartridges covering centrifugal microfluidic unit operations, and exchangeable tools for robotic unit operations. The latter enables magnetic bead manipulation, as well as in-rotation liquid supply and valve actuation through synchronisation of cartridge rotor and tool rotor.

The architecture of *RoCM* relies on the synchronised interplay of two main groups of components: exchangeable cartridges for microfluidic operations and exchangeable tools for robotic operations (Figure 1). A key function of this architecture is the capability of synchronised co-rotation of microfluidic cartridges and pipetting-tools, an operation hereinafter referred to as in-rotation liquid supply [40]. The following elements are required for a *RoCM* system:

**Robotic arm:** The robotic arm is the central automation element. A 4-axis robotic arm has been chosen due to its high workspace-to-footprint ratio and expandability, enabled by preinstalled signal lines for future tool integration. Notably, depending on cost and space requirements, other automation techniques, such as X-Y-Z-stages, could be used instead of a robotic arm.

**Tool rotor with tool changer:** For liquid transfer onto a rotating cartridge, we opted for co-rotation of the pipette tip with the rotating cartridge over a static droplet dispenser [41], since the latter is less efficient for up to millilitre-scale volumes. To achieve co-rotation, we opted for a tool rotor with a slip ring connected to a tool changer. This concept allows the robotic arm to position statically above the cartridge rotor while only the tool changer itself rotates (Figure 1). The tool changer is designed to pick up individual tools as needed and secures them through a mechanical locking mechanism.

**Cartridge rotor:** The cartridge rotor fixes the cartridge through vacuum suction, provides centrifugal forces, and is equipped with integrated Peltier elements, enabling temperature control through thermoelectric heating and cooling.

**Exchangeable cartridges:** The cartridges encompass well-established centrifugal microfluidic unit operations [42] such as mixing, incubation, bead handling, filtration, liquid transfer, and metering, alongside a novel monolithically integrated valving concept [43] actuated via synchronised robotic liquid supply.

**Figure 2:**
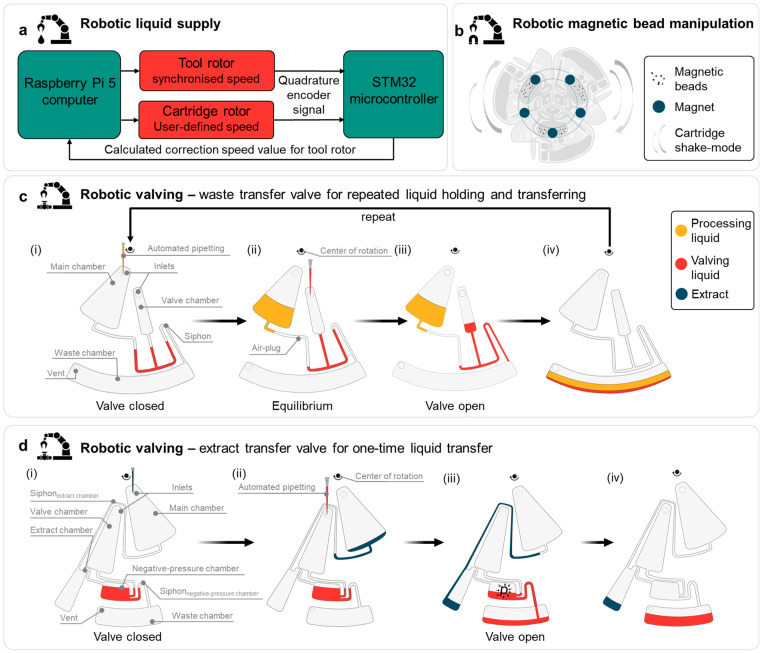
**Robotic unit operations.** (**a**) Scheme for synchronised rotation of the tool rotor relative to the preprogrammed speed of the cartridge rotor; (**b**) top view of a magnet constellation (blue) in the magnetic tool over three assembled cartridges; (**c**) scheme for robotic valving, which can be used as a waste transfer valve for repeated liquid holding and transferring. The main chamber with inlet (radially most inward) is the incubation chamber in an extraction protocol. The valve chamber has an inlet positioned radially, further outward. Red represents valving liquids (e.g., water), yellow represents processing liquids (e.g., plasma and subsequent buffers). An enclosed air-plug assures a stable equilibrium, e.g., during incubation of buffers; (**d**) schematic of robotic valving as an extract transfer valve for one-time liquid transfer. Blue represents the extract, e.g., a cfDNA eluate. Compared to the waste transfer valve application, the extract transfer valve can transfer the desired liquid (here: extract) without it getting into contact with the valving liquid. Valving steps: (i) the valve is prepared by initially filling the negative-pressure chamber with valving liquid. (ii) Then the extract is added to the main chamber. To actuate the transfer of the extract to its extract chamber, the negative-pressure chamber is further filled to overcome the siphon_negative-pressure chamber_. (iii) Upon partial emptying of the negative-pressure chamber, the negative pressure is generated in the extract chamber, which sucks the extract from the main chamber over the siphon_extract chamber_. From there, the extract is pumped radially outwards to the extract chamber. (iv) At the end of this process, both extract and waste chamber should be filled with the extract from the main chamber and the valving liquid from the negative-pressure chamber, respectively.

**Exchangeable tools:** Using pipette tools and a magnet tool, we established three key unit operations critical for automation of most extraction protocols: liquid supply (Figure 2a), magnetic bead manipulation (Figure 2b), and valving (Figure 2c,d). The modular system architecture allows further extension with other tools if required, for example, optical detectors.

#### 3.1.2. RoCM Unit Operations

Automation of multi-analyte liquid biopsy protocols required the development and characterisation of three new *RoCM* operations based on the interplay of robotics and microfluidics.

**Robotic liquid supply:** To synchronise the tool rotor for liquid supply, both the cartridge rotor and tool rotor transmit quadrature encoder signals to an STM32 microcontroller (Figure 2a). The microcontroller calculates the correction speed value for the tool rotor and transmits it to the Raspberry Pi single-board computer, which communicates with both the cartridge rotor and tool rotor, adjusting the latter accordingly.

**Robotic magnetic bead manipulation:** To enable efficient mixing and dispersion of the magnetic beads during binding, washing and eluting, e.g., during cfDNA extraction, the cartridge containing the beads is repeatedly accelerated and decelerated within a stationary magnetic field. This field is generated by five permanent magnets arranged evenly in a concentric pattern and held by a 3D-printed magnetic tool (Figure 1). During bead-mixing, the robotic arm positions the stationary tool above the rotating cartridge, which undergoes consecutive acceleration and deceleration cycles to promote bead agitation and homogenous dispersion (Figure 2b).

**Robotic valving:** In this system, in-rotation liquid supply serves a dual function, acting as the reagent input to the main chamber as well as the input for the valving liquid to control individual valves, each of which is addressed through its dedicated inlet. This architecture enables precise and programmable control over liquid routing, as illustrated in Figure 2c. Robotic valving is achieved in four steps [43]:

(i) First, the valve is closed with valving liquid. (ii) When pipetting processing liquid into the main chamber, a stable air-plug is trapped inside the connecting channel between the valving and the processing liquid, allowing retention of volumes in the millilitre range inside the main chamber. (iii) Upon further addition of valving liquid, the siphon is primed, initiating valve opening. (iv) In the final state, controlled radial outflow of the processing liquid from the main chamber into the waste chamber is realised. The valve can subsequently be closed again by refilling the valving unit, allowing the process to be repeated as needed. This is particularly useful for multiple washing steps while, e.g., magnetic beads for binding remain in the main chamber. The specific design of the outlet channels at the radial most outward position enables valving without any noticeable residual liquid. A second implementation of robotic valving, designed for single use and ensuring that the processing liquid does not come into contact with the valving liquid (e.g., for final extract transfer), is described in Figure 2d.

#### 3.1.3. Design of Microfluidic Cartridges for cfDNA and EV Extraction Using RocM

To demonstrate the capabilities of the *RoCM* platform and its novel unit operations, we designed two dedicated cartridge layouts for the extraction of cfDNA and EVs from plasma. These analytes represent two clinically interesting analytes with distinct biophysical properties and extraction requirements.

**Figure 3:**
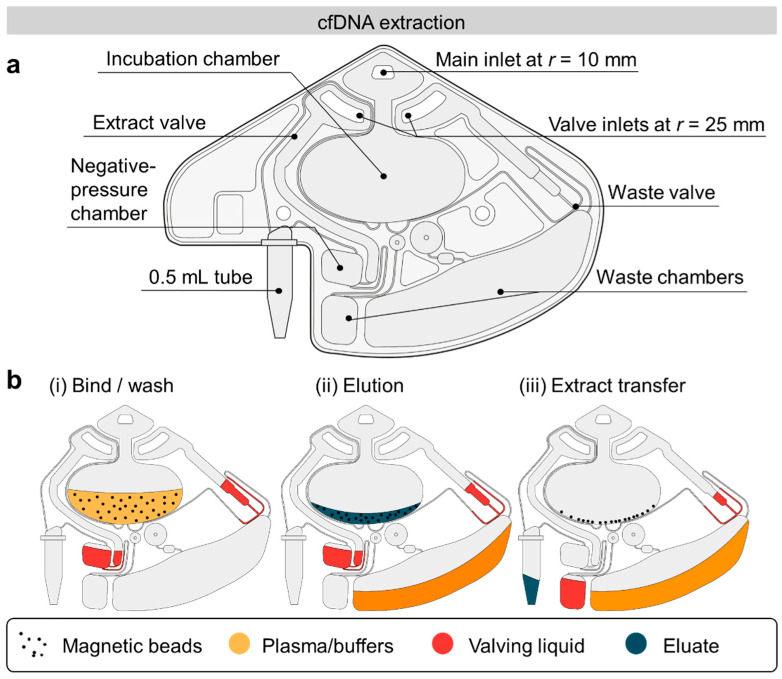
**Microfluidic cartridge design for cfDNA extraction.** (**a**) Microfluidic design of RoCM-cfDNA slice; (**b**) steps of cfDNA bind–wash–elute protocol, including (i) binding of cfDNA from plasma to magnetic silica beads, followed by washing steps, (ii) eluting cfDNA from beads using an elution buffer (blue), and (iii) transferring eluate (blue) to the attached tube.

The *RoCM-cfDNA slice* (Figure 3a) was developed to integrate a magnetic bead-based bind–wash–elute protocol for processing 1 mL of plasma.

The central incubation chamber functions as the main reaction site, where silica-coated magnetic beads realise cfDNA capture and purification. From this chamber, liquids are completely valved to one of two downstream paths: either to the waste chamber or to the elution tube, in the final step (Figure 3b). Both valve channels are placed at the radially outermost positions of the incubation chamber, preventing residual volumes from remaining in the chamber. Robotic valves are actuated according to the principles shown in Figure 2c,d.

**Figure 4:**
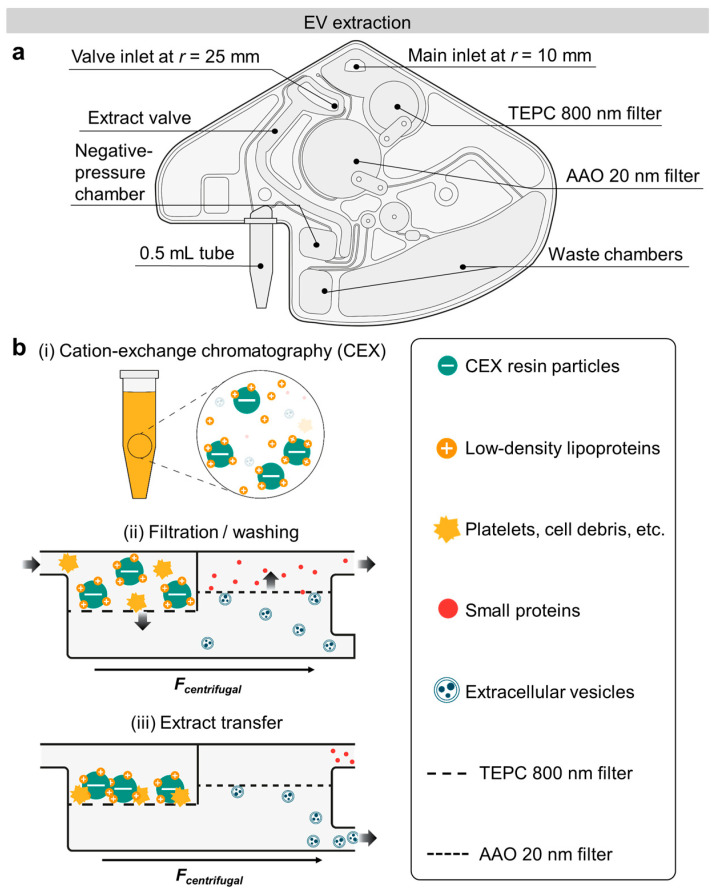
**Microfluidic cartridge design for EV extraction.** (**a**) Microfluidic design of RoCM-EV slice; (**b**) steps of EV extraction protocol, including (i) off-cartridge incubation with cation-exchange chromatography (CEX) resin, (ii) filtration and washing, and (iii) extract transfer to the attached tube. TEPC: track-etched polycarbonate; AAO: anodic aluminium oxide.

The *RoCM-EV slice* (Figure 4a) was used for the processing of 400 µL of plasma and is adapted from a protocol presented by Arjmand and colleagues [44].

The steps for EV extraction include (i) initial binding of, e.g., undesired positively charged lipoproteins by cation-exchange chromatography (CEX) resin (Figure 4b). (ii) The *RoCM-EV slice* applies a sequential filtration approach, based on two filter chambers with membranes at pore sizes of 800 nm and 20 nm, respectively. Following sample filtration, the sample is washed with 1 mL PBS, which also serves as the extraction medium. The first filter retains cell debris, protein aggregates, and CEX resin particles [44], the second filter retains the EVs. (iii) At the second filter chamber, the waste channel is positioned to discard volumes exceeding 100 µL (filtrate), ensuring that 100 µL of the EV concentrate (retentate) in PBS is completely valved into the collection tube, which is actuated by a robotic valve at the end of the process, analogous to the *RoCM-cfDNA slice* (Figure 2d).

#### 3.1.4. Automated RoCM Protocols

As with other automation solutions, several preparatory steps are performed manually, prior to initiating the automated workflow. These include (i) assembling up to three injection-moulded cartridges using pressure-sensitive adhesive foil (9795R, 3M) before placement onto the vacuum zones of the rotor (Supplementary Figure S1a), (ii) attaching 0.5 mL tubes to the cartridge, which is designed to be leak-tight without additional sealing materials, and (iii) preparing reagents in the tube rack and opening tube lids (Supplementary Figure S1b). From this point, all subsequent steps are automated. A representative part of the sequence can be viewed in Supplementary Video S1. As an example, the robotic arm first equips itself with a pipetting tool configured for the *r* = 25 mm radius, picks up a pipette tip, and loads water into the valving units. Centrifugation at high frequency (e.g., 40 Hz) pins the liquid to close the valves. The tip is then discarded, and the tool is exchanged for a second pipetting tool for rotation at *r* = 10 mm, enabling sample and buffer loading into the incubation chamber. For magnetic bead-based protocols, as in the case of cfDNA extraction, the robotic arm engages the magnetic tool and positions it above the incubation chamber, while the cartridge rotor initiates a shake-mode [45], e.g., between 3 and 20 Hz at 20 Hz/s, to facilitate effective mixing. Ultimately, for the two demonstrated cartridge types, the extracted analytes are valved into a tube press-fitted to the cartridges. After stopping the rotation, the user removes the tubes from the cartridges to receive purified analytes for downstream analyses.

### 3.2. Characterisation of Robotic Unit Operations

#### 3.2.1. Robotic Liquid Supply

**Figure 5:**
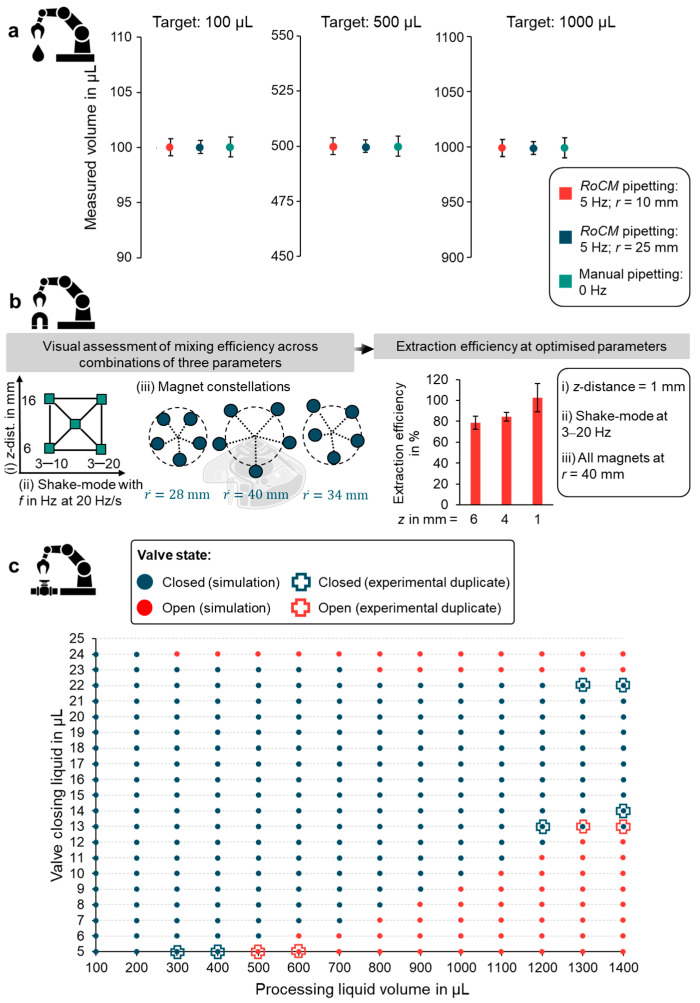
**Characterisation of robotic unit operations.** (**a**) Assessment of manual pipetting (0 Hz) vs. RoCM pipetting under rotation (5 Hz) at radii of 10 mm and 25 mm using water; (**b**) optimisation of binding efficiency through parameter testing of magnetic actuation. Mixing efficiency was qualitatively assessed from recorded videos by three independent evaluators, based on visible bead movement and redistribution (Video S3); (**c**) simulated and experimental operation window of waste transfer valve actuation in a test cartridge, covering the valve’s operational range from 3 to 40 Hz with accelerations of 2–20 Hz/s. Only selected boundary conditions were experimentally tested, with intermediate values assumed to fall within the validated working range. Experimental data points were considered valid only when both replicates yielded identical outcomes (valve open or closed).

As pipetting accuracy is a matter of pipette calibration, we focused on evaluating the precision of *RoCM* pipetting under rotation. A frequency of 5 Hz was chosen as a compromise between stability and safety. At this speed, the centrifugal field is sufficient to counteract capillary effects that would otherwise, for example, cause wetting liquids in the waste chamber to creep radially inward into the waste transfer valve (an effect observed when pipetting was performed under static conditions). Frequencies > 5 Hz were not tested, to avoid excessive inertial loads and potential collision between the mounted pipette and the system periphery. For qualitative evaluation of robotic liquid transfer under 5 Hz rotation, the recording can be seen in slow motion in Supplementary Video S2. To assess pipetting reproducibility, we conducted volumetric tests (100, 500 and 1000 µL) with a test cartridge (Supplementary Figure S3). We compared manual pipetting of water at standstill (0 Hz) against *RoCM* pipetting under rotation (5 Hz) at radii of 10 mm and 25 mm, each measured in triplicate (Figure 5a). The coefficient of variation (CV) remained below 0.9% across all conditions, indicating comparable precision between manual and *RoCM* pipetting. These results demonstrate that pipetting under rotation at 5 Hz maintains accuracy and precision without being compromised by centrifugal motion. This is critical, particularly given that the *RoCM-cfDNA* and *EV* extraction workflows require a total of 20 and 6 pipetting steps, respectively.

#### 3.2.2. Magnetic Bead Manipulation

To optimise cfDNA extraction, we compared the *RoCM-cfDNA slice* and a manual bead-based extraction, using real-time quantitative PCR (qPCR). As artificial samples, we spiked nucleosomes from cancer cell lines with a known *KRAS* G13D mutation into plasma from healthy donors, mimicking circulating tumour DNA (ctDNA_surrogate_) [46]. Each on-cartridge step was individually tested by performing single steps on-cartridge while executing other steps manually. Preliminary results (Supplementary Figure S4) identified the binding step as the most critical. To optimise robotic microfluidic mixing efficiency during binding, we investigated three key parameters: (i) *z*-distances of 6, 11, and 16 mm between the cartridge and the five magnets; (ii) shake-mode frequencies at 20 Hz/s acceleration/ deceleration (3–10 Hz or 3–20 Hz); and (iii) magnet constellation (all magnets at *r* = 28 mm, all at *r* = 40 mm, or in an escalating pattern with mean *r* = 34 mm) to vary the radial range of bead actuation within the cartridge (Figure 5b). Mixing behaviour was assessed by qualitative analysis of stroboscopic image sequences by three individual evaluators (Supplementary Video S3). Visual assessment refers to a comparative, frame-by-frame evaluation of bead redistribution dynamics, focusing on the rate of bead dispersion and spatial homogeneity across the mixing chamber, over time. Temporal changes in pixel intensity patterns between successive frames served as a proxy for bead motion and redistribution, without extracting an absolute mixing metric. Based on this parameter screening, the *z*-distance appeared to have the strongest effect on bead mixing. Fixing the shake-mode (3–20 Hz) and magnet constellation (all at *r* = 40 mm), we tested different *z*-distances, and found that 1 mm yielded the highest extraction efficiency of 102.64% (±13.50%), compared to manual reference using spiked plasma (Figure 5b).

#### 3.2.3. Robotic Valving

To demonstrate and characterise the robotic microfluidic waste transfer valve, we conducted a concept study using a dedicated test cartridge with a defined radial position (Supplementary Figure S5). The test design allowed a maximum liquid volume of 1400 µL inside the main chamber. A centrifugal microfluidic simulation model [36] was employed to simulate the valve’s operation window over a frequency range of 3–40 Hz with accelerations of 2–20 Hz/s, in order to evaluate its tolerance against parameter changes (Figure 5c). Selected conditions were experimentally evaluated in technical duplicates using water. The results confirmed that a valve closing liquid volume of, e.g., 14 µL only, could hold up to 1400 µL of processing liquid. Based on this experimentally verified simulation, valve placement and operating conditions for application-specific cartridges at different radial positions were derived. The *RoCM-cfDNA slice* design was adapted to hold at least 3700 µL of processing liquid (plasma, proteinase K, lysis buffer, and binding buffer), with a 20 µL valving liquid that withstands the required frequency protocols. Detailed protocols of the *RoCM-cfDNA* and *RoCM-EV slice* can be found in Supplementary Tables S3 and S5.

### 3.3. Demonstration of RoCM Using Patient Samples

Following preliminary evaluation using spiked plasma samples (Supplementary Figure S6), the *RoCM* platform was applied in two independent clinical settings: *RoCM-cfDNA slices* were used to process plasma samples from colorectal cancer (CRC), multiple myeloma (MM) and melanoma patients at the Department of Hematology and Oncology (University Medical Center Schleswig-Holstein, Lübeck), which specialised in cfDNA-based diagnostics, whereas *RoCM-EV slices* were applied to plasma samples from prostate cancer patients at the Institute for Infection Prevention and Control (Medical Center, Freiburg), which focuses on EV biomarker analysis.

#### 3.3.1. RoCM-cfDNA Slices

To demonstrate the applicability and performance of the *RoCM-cfDNA slices*, plasma from one melanoma, three CRC, and two MM patients with known mutations (determined via tissue sequencing) were analysed. Each sample was split into 1 mL plasma aliquots and co-extracted using a commercial robotic instrument (EZ2, QIAGEN). If sufficient material was available, additional 1 mL aliquots were either processed as replicates or co-extracted using a manual column-based approach with the QIAamp Circulating Nucleic Acid Kit (QIAGEN) [47]. All methods resulted in an eluate of 100 µL, which was analysed using a 7-plex digital PCR (dPCR) assay [37] targeting *BRAF* wild-type (WT), V600E, V600K and *NRAS* WT, Q61K, Q61L, and Q61R. To compare extraction performance, WT copy numbers obtained with the *RoCM-cfDNA slices* were normalised to those obtained with the EZ2 extraction from the same plasma sample, which was used as a reference and defined as 100% extraction efficiency. The mean WT recovery of the *RoCM-cfDNA slices* was determined as 40.0% (±12.8%) of the EZ2 results across eleven extractions.

**Figure 6:**
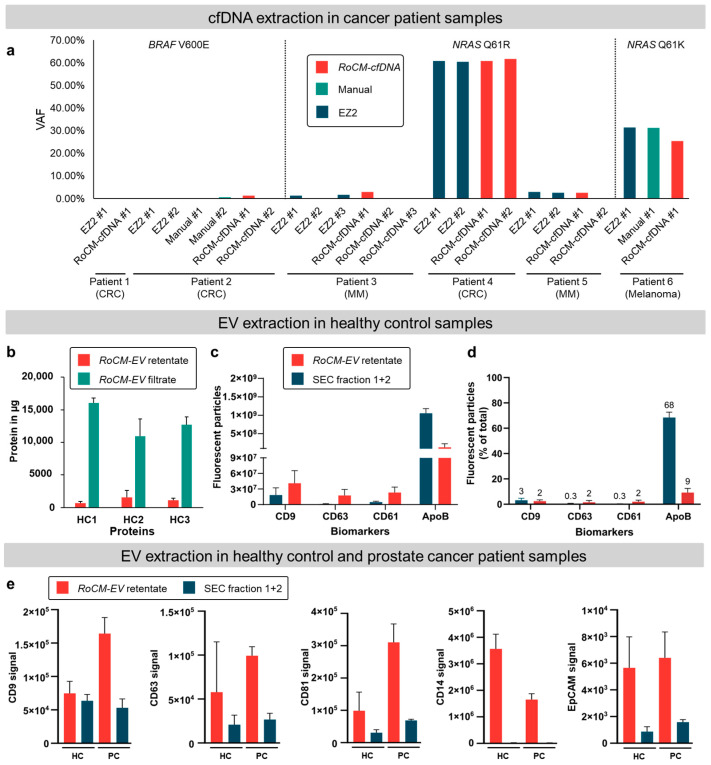
**Demonstration of RoCM using patient samples.** (**a**) Comparison of VAF across extraction methods. The x-axis represents six patients, each extracted manually, using the EZ2 device, or RoCM-cfDNA slices, with replicates when a sample was available. Each data points corresponds to the mean of a dPCR duplicate run; (**b**) volume-adjusted protein quantification of retentate (EV concentrate) and filtrate (waste) samples of three healthy controls, each measured in technical triplicates; (**c**) NanoFCM-based and volume-adjusted biomarker quantification in RoCM-EV retentates compared to SEC pooled fraction 1 and 2. Standard deviations derive from three healthy control samples; (**d**) the percentage of fluorescent particles was calculated relative to the total particle count measured in scatter mode (used as the reference for 100% of detected particles). Standard deviations derive from three healthy control samples; (**e**) MSD-based biomarker measurement of healthy controls and prostate cancer patients, each in triplicates. MSD: Meso Scale Discovery; #: replicate, VAF: variant allele frequency; CRC: colorectal cancer; MM: multiple myeloma; HC: healthy control; PC: prostate cancer patient.

However, when variant allele frequencies (VAFs), a clinically important parameter, were calculated for the clinical samples as the ratio of mutant copies to total (mutant + WT), the determined VAFs show good concordance among the three methods (Figure 6a). When patients had a low VAF (<3%), occasional dropouts occurred randomly across all three methods, without any method being particularly prone to failure. This suggests that the observed variability is likely due to stochastic effects arising from the low number of mutant copies available for detection.

#### 3.3.2. RoCM-EV Slices

First, bulk protein quantification, used as an indicator for purity, revealed an average protein clearance of 91.9% (±4.3%) by *RoCM-EV slices*, calculated as the ratio of the protein amount in the filter retentate to the total (sum of filtrate and retentate) protein amount (Figure 6b).

To assess the biological diversity and purity of the isolated EVs, a panel of four biomarkers was selected to represent distinct EV origins and potential contaminants. CD9 and CD63 were chosen as canonical tetraspanins and universal EV markers, according to MISEV guidelines [48], while CD61 (integrin β-3) was included to identify platelet-derived EVs, which are known to differ in size and density from other EV subpopulations [49]. ApoB was incorporated as a marker for undesired lipoprotein co-isolation [50], providing an estimate of sample purity relative to EV-associated signals. Biomarker expression in the retentate was then evaluated by both single-EV and bulk-EV analyses. Single-EV analysis was performed using nano-flow cytometry (NanoFCM) to quantify the presence of EV-associated (CD9, CD63, and CD61) and lipoprotein-associated (ApoB) markers (Figure 6c and Supplementary Figures S7–S12). Particle counts revealed that isolation by *RoCM-EV* resulted in higher numbers of particles positive for EV markers CD9, CD63, and CD61, compared to SEC. Conversely, particles positive for the undesired lipoprotein marker ApoB were approximately threefold lower in the *RoCM-EV*-isolated samples (Figure 6c). The stronger overall signal observed for ApoB compared with EV markers is consistent with the substantially higher abundance of lipoproteins in plasma, as discussed by Simonsen [50].

When the number of fluorescent particles was normalised to the total particle count measured in scatter mode (Figure 6d), both isolation methods showed overall low proportions (0.3–3%) of EV marker-positive events. Notably, the percentage of CD9-positive particles was comparable between the two methods, whereas the percentages of CD63- and CD61-positive particles were approximately one order of magnitude higher following *RoCM-EV slice* isolation, compared to SEC. In contrast, ApoB-positive particles, indicating an undesired contamination with lipoproteins, decreased markedly, from 68% with SEC to 9% with the *RoCM-EV*-based method.

To evaluate the performance of *RoCM-EV* in healthy and cancer-derived samples, we conducted a multiplexed immunoassay using the Meso Scale Discovery (MSD) platform. EV biomarker signals were quantified from the *RoCM-EV slice* retentate, which contains the enriched EV fraction (Figure 6e), and compared to pooled SEC fractions 1 and 2, representing the EV-rich eluate. For completeness, signals from the corresponding filtrate are provided in the Supplementary Information (Supplementary Figure S13). Samples included EV-enriched fractions from three healthy control donors (HC) and three prostate cancer patients (PC). The panel of markers included universal EV biomarkers (CD9, CD63, and CD81), the monocyte/macrophage marker CD14, and EpCAM, an epithelial marker commonly overexpressed in cancer [51]. To account for EV heterogeneity and the variable epitope density per vesicle, results were reported in signal intensities, rather than absolute concentrations. All assays were performed using 50 µL of undiluted sample. Notably, signals for CD9, CD81 and CD63 yielded consistently higher signals in the *RoCM-EV slice* retentate, compared to SEC-isolated fractions.

To assess the recovery of immune-cell-derived EVs, we additionally analysed CD14 expression, which has been reported to serve as biomarker of inflammation, thrombosis and tumour spread/burden [52]. The results showed a clear difference between *RoCM-EV* and SEC fractions 1 + 2 in the EV distribution at the isolation level. While the two SEC fractions yielded negligible signal, CD14-positive EVs were clearly detectable in both *RoCM-EV* retentate (Figure 6e) and filtrate (Supplementary Figure S13). This immune-related EV subpopulation is likely present in later SEC fractions, which were not included in this comparison.

Evaluation of the epithelial cancer marker, EpCAM, revealed a similar trend. *RoCM-EV* retentates produced signals approximately one order of magnitude higher than those obtained from SEC-isolated fractions 1 + 2, further confirming the platform’s capacity to enrich EVs of diverse cellular origin.

Collectively, these findings demonstrate that *RoCM-EV* enables the isolation of a broader and more representative EV subpopulation compared to conventional SEC, while reducing the contamination by co-isolated ApoB-positive lipoproteins. *RoCM-EV* further concentrates the EVs into smaller extraction volumes (100 µL concentrate vs. 400 µL per SEC fraction), reducing dilution effects during downstream analysis.

## 4. Discussion

A key function of the *RoCM* architecture is the in-rotation liquid supply. It ensures precise fluid control and valving, without losing control of liquid manipulation via centrifugal forces. This is particularly crucial for wetting liquids such as alcohol buffers, to prevent unwanted capillary effects that can cause uneven liquid distribution, uncontrolled spreading, or retention in undesired areas. This continuous rotational operation preserves hydrostatic stability and prevents pressure fluctuations that typically cause bubble formation in conventional stop-and-go actuation schemes. Additionally, in-rotation liquid supply eliminates the need for large pre-loaded liquid reservoirs on the cartridge, saving valuable footprint.

***RoCM-cfDNA slices*:** for protocol optimisation, robotic handling was initially performed at a safety-limited speed, resulting in a total run time of 135 min for processing three *RoCM-cfDNA slices* in parallel (Supplementary Table S6). Upon speed optimisation of robotic handling, the runtime can be significantly reduced, to become comparable to the manual bead-based protocol (90 min).

The reduced mean WT recovery from clinical plasma samples, compared to the preliminary evaluation with spiked plasma samples (Supplementary Figure S6A) may result from matrix-related effects, rather than being attributed to the actuation principle itself. Clinical plasma samples typically contain substantially lower cfDNA concentrations and a more complex matrix composition than artificially spiked samples, which is known to affect bead–analyte binding efficiency and washing performance [53]. The binding conditions were applied without any re-optimisation to clinical material. Accordingly, the observed recovery reflects the current protocol implementation within the *RoCM* workflow, rather than an inherent limitation of the automated platform architecture. Further refinement of the bead mixing and binding steps (Supplementary Figure S4), using patient samples, is expected to improve recovery.

***RoCM-EV slices:*** further optimization of EV recovery based on spiked material (Supplementary Figure S6B), e.g., via use of surface blocking solutions, is hypothesised to help minimise EV binding to COC surfaces and filters inside *RoCM-EV slices*, reducing loss and improving overall recovery. This optimisation is currently under investigation as a follow-up to the recent publication by Arjmand and colleagues [44]. The total run time was 80 min for processing three *RoCM-EV slices* in parallel. As a comparison, SEC requires roughly 85 min to process a single sample, plus an additional 5 min per extra sample, including column preparation, fractionation and washing (Supplementary Table S7).

Regarding the clinical plasma samples, the residual protein content in the retentate was in the same order of magnitude as values reported by another group employing filtration cartridges with a pore size of 20 nm [31]. However, the improved purity observed with our system is likely due to the preceding incubation with CEX resin, which exploits differences in surface charge between lipoproteins and EVs. While EVs are preferentially retained under the applied conditions, a substantial fraction of ApoB-containing lipoproteins binds to the CEX resin and is consequently depleted. These results confirm that the incorporation of a charge-based separation step substantially reduces co-isolated lipoproteins, while enriching EV-associated markers. This is consistent with previous reports, highlighting the need for orthogonal purification strategies [54,55].

**Valving**: regarding the valving concept, our system employs monolithically integrated valves directly fabricated into the cartridge structure. This approach eliminates the need for additional fabrication complexity (3D-printed push-and-twist mechanisms and elastic diaphragm bonding) as required by similar centrifugal microfluidic platforms [56], while still enabling repeated liquid holding and transferring within the cartridge [43].

**Flexibility and sample parallelisation:** in contrast to existing robotic handling workstations or microfluidic systems, which rely on single-extraction modality, such as bead-based affinity capture or dielectric separation, *RoCM* offers functional flexibility. It supports multiple operation modes determined by the cartridge design, actuators picked up by the robotic arm (e.g., pipettes and magnets), and the rotor on which the cartridge is mounted (e.g., with integrated heating and cooling zones). Compared to previously reported centrifugal microfluidic platforms [29,32], *RoCM* achieves a higher degree of sample parallelisation, as reagents are supplied robotically on demand, rather than being pre-stored on the cartridge. In the configuration presented in this work, *RoCM* can simultaneously process three plasma samples of 1000 µL each for cfDNA extraction or three samples of 400 µL each for EV extraction, which represents the current maximum parallel capacity of the system. The number of parallel samples or sample volumes can theoretically be increased by larger rotor size or multiple rotors underneath a single robotic arm.

The present study constitutes a proof-of-concept demonstration of the RoCM platform. While the system-level architecture introduces novel capabilities, including in-rotation liquid handling and flexible workflow integration, the demonstrated applications remain at an early stage. Accordingly, evaluations are based on limited sample numbers and technical replicates, and are therefore primarily descriptive. While trends were consistent, larger cohorts and systematic replication will be required, to enable comprehensive statistical validation. Importantly, the fluid handling performance was found to be comparable to manual pipetting under the tested conditions, indicating that the primary advantage of the system lies in automated workflow integration, reproducibility, and sample parallelisation, rather than in enhanced performance of individual unit operations.

## 5. Conclusions

We introduce Robotic Centrifugal Microfluidics, *RoCM*, a multi-functional, benchtop-sized platform designed for automation of versatile protocols, such as isolation of different analytes from liquid biopsies with increased sample parallelisation. The unique architecture of *RoCM* combines centrifugal microfluidics with robotic liquid handling, enabling automation of highly integrated cartridges for increasingly complex protocols. Novel unit operations, e.g., in-rotation liquid supply and robotic actuation of microfluidic valves, enable long protocols on a small cartridge footprint. As a proof-of-concept, we demonstrate the extraction of cfDNA and EVs from blood plasma samples, two analytes that require orthogonally distinct extraction modes. Experiments with spiked and clinical plasma samples confirm the capability of *RoCM* of achieving comparable results as reference manual and automation methods, while integrating both protocols into a single instrument. Using the suggested components, the robotic system remains comparable in costs to other automated liquid handlers (40,000–60,000 EUR), avoiding high financial barriers for academic and translational research laboratories. Broad adoption will depend largely on cartridge cost. Our demonstration of injection-moulded cartridges represents a key step towards scalable and affordable consumables.

With a robotic arm capable of tool exchange and a broad repertoire of established centrifugal microfluidic unit operations, the *RoCM* platform is primed for additional functions, e.g., optical detectors for integrated readout, incorporating dedicated pick-and-place mechanisms for fully automated cartridge handling, or previously demonstrated on-cartridge blood plasma separation [36]. The *RoCM* platform further allows scalability in sample parallelisation, for example, by operating multiple rotors actuated by the same robotic arm. Its principle of exchangeable microfluidic cartridges allows the system to be further extended to other protocols, such as isolation of CTCs, chromatin immunoprecipitation (ChIP), methylated DNA immunoprecipitation (MeDIP), or next-generation sequencing (NGS) library preparation. Beyond these sample preparation applications, *RoCM* offers broad applicability across disciplines that benefit from programmable, reconfigurable or real-time microfluidic workflows. Its flexibility makes it particularly well-suited for laboratories engaged in method development, where protocols evolve continuously. Potential applications include high-throughput nanoparticle synthesis [57], where centrifugal devices have recently shown promise, and 3D cell culture platforms [58], where *RoCM* could support automated media exchange and compound dosing for drug sensitivity testing.

## 6. Patents

Peter Juelg, Yumi Kaku, Moritz Bösenberg, Nils Paust, Tobias Hutzenlaub and Jan Lüddecke are co-inventors on the European patent application *Liquid transfer between rotating modules* (EP4483190A1; filed 7 February 2023), which describes a device enabling synchronised rotation of a fluidic module and a transfer module to allow controlled liquid transfer between rotating modules. This concept forms the basis for **in-rotation liquid supply** in centrifugal microfluidic systems. Truong-Tu Truong, Peter Juelg, Yumi Kaku, Judith Schlanderer, Nils Paust and Tobias Hutzenlaub are co-inventors on the patent application *Retention and Transfer of Liquids* (PCT/EP2024/072046; filed 7 August 2023), which builds on this approach and utilises in-rotation liquid supply to realise a **liquid-actuated valving mechanism**.

## Data Availability

All data used in this article have been included in the cited works.

## Supplementary Materials

The following supporting information can be downloaded at: https://www.mdpi.com/article/10.3390/bios16060309/s1, Figure S1: Technical implementation of RoCM concept; Figure S2: Channel dimensions of cartridge designs; Figure S3: Milled PMMA cartridge for volumetric pipetting tests; Figure S4: Preliminary optimisation of the RoCM-cfDNA extraction workflow; Figure S5: COC foil cartridge for evaluation of robotic valve actuation for repeated liquid holding and transfer; Table S1: Decision matrix for concept with rotating SCARA; Table S2: Decision matrix for concept with rotating Cartesian robot; Table S3: RoCM-cfDNA slice extraction protocol; Table S4: Reaction mixes and PCR conditions for both PCR platforms; Table S5: RoCM-EV slice filtration protocol; Figure S6: Preliminary evaluation of RoCM using spiked plasma samples; Figures S7–S12: Single EV analysis using NanoFCM; Figure S13: Demonstration of RoCM-EV slices using patient samples; Table S6: Comparison of the three cfDNA extraction methods; Table S7: Comparison of the two EV extraction methods; Video S1: representative part of the protocol sequence; Video S2: In-rotation liquid supply in slow-motion; Video S3: Bead mixing behaviour.

## Abbreviations

The following abbreviations are used in this manuscript:

RoCM	Robotic Centrifugal Microfluidics
cfDNA	Cell-free DNA
EV(s)	Extracellular vesicle(s)
CTC(s)	Circulating tumour cell(s)
CRC	Colorectal cancer
MM	Multiple myeloma
VAF	Variant allele frequency
WT	Wild-type
SEC	Size/exclusion chromatography
CEX	Cation-exchange chromatography
(D)PBS	(Dulbecco’s) phosphate-buffered saline
MSD	Meso Scale Discovery
NanoFCM	Nano-flow cytometry
ApoB	Apolipoprotein B
EpCAM	Epithelial cell adhesion molecule
MISEV	Minimal Information for Studies of Extracellular Vesicles
DMF	Digital microfluidics
qPCR	Quantitative PCR
dPCR	Digital PCR
ctDNA	Circulating tumour DNA
CAD	Computer-aided design
PMMA	Poly (methyl methacrylate)
COC	Cyclic olefin copolymer
PE	Polyethylene
AAO	Anodic aluminium oxide
TEPC	Track-etched polycarbonate
BoPET	Biaxially oriented polyethylene terephthalate
F-NTA	Fluorescent nanoparticle tracking analysis
SCARA	Selective Compliance Assembly Robot Arm
ChIP	Chromatin immunoprecipitation
MeDIP	Methylated DNA immunoprecipitation
NGS	Next-generation sequencing
HC	Healthy control
PC	Prostate cancer
SS	Side scatter
QC	Quality control
