# Supplementary material for: Robotic Centrifugal Microfluidics with In-Rotation Liquid Supply for the Extraction of Multiple Liquid Biopsy Analytes in One Platform
Source: Biosensors (Basel). 2026 May 28;16(6):309. doi: 10.3390/bios16060309 (PMC13296457; doi:10.3390/bios16060309)
Supplement: Supplementary file 1 [file biosensors-16-00309-s001.zip › biosensors-4243775-supplementary.pdf]

Supplementary Information

Robotic Centrifugal Microfluidics with In-Rotation Liquid Supply for the Extraction of Multiple Liquid Biopsy Analytes in One Platform

Truong-Tu Truong [1,*,†], Yumi Kaku [1,†], Gonzalo Bustos-Quevedo [2,3], Sara ElGenk [1], Ehsan Mahmodi Arjmand [1], Gustav Grether [1], Jan Lüddecke [1], Judith Schlanderer [1], Stefan Wagner [4], Theresa Katschmareck [5,6], Eva Dazert [5,6], Nikolas von Bubnoff [5,6,7], Irina Nazarenko [1,2,3], Germán Matías Hansen [1], Sabrina Kartmann [1,8], Tobias Hutzenlaub [1,7,8], Nils Paust [1,7,8] and Peter Juelg [1,7,8]

[1] Hahn-Schickard, 79110 Freiburg, Germany; ehsan.arjmand@hahn-schickard.de (E.M.A.); gustav.grether@hahn-schickard.de (G.G.); jan.lueddecke@hahn-schickard.de (J.L.); judith.schlanderer@hahn-schickard.de (J.S.); irina.nazarenko@uniklinik-freiburg.de (I.N.); matias.hansen@hahn-schickard.de (G.M.H.); sabrina.kartmann@hahn-schickard.de (S.K.); tobias.hutzenlaub@hahn-schickard.de (T.H.); nils.paust@hahn-schickard.de (N.P.); peter.juelg@hahn-schickard.de (P.J.)

[2] Institute for Infection Prevention and Control, Medical Center, Faculty of Medicine, 79106 Freiburg, Germany; gonzalo.salvador.bustos.quevedo@uniklinik-freiburg.de

[3] Faculty of Biology, University of Freiburg, 79104 Freiburg, Germany

[4] Hahn-Schickard, 70569 Stuttgart, Germany; stefan.wagner@hahn-schickard.de

[5] Department of Hematology and Oncology, University Medical Center Schleswig-Holstein, Campus Lübeck, 23538 Lübeck, Germany; theresalilith.katschmareck2@uksh.de (T.K.); eva.dazert-klebsattel@uksh.de (E.D.); nikolaschristiancornelius.vonbubnoff@uksh.de (N.v.B.)

[6] University Cancer Center Schleswig-Holstein, University Medical Center Schleswig-Holstein, Campus Lübeck, 23538 Lübeck, Germany

[7] European Liquid Biopsy Society (ELBS), 20246 Hamburg, Germany

[8] Laboratory for MEMS Applications, IMTEK—Department of Microsystems Engineering, University of Freiburg, 79110 Freiburg, Germany

* Correspondence: tu.truong@imtek.uni-freiburg.de

† These authors contributed equally to this work.

System architecture and technical implementation of the RoCM platform

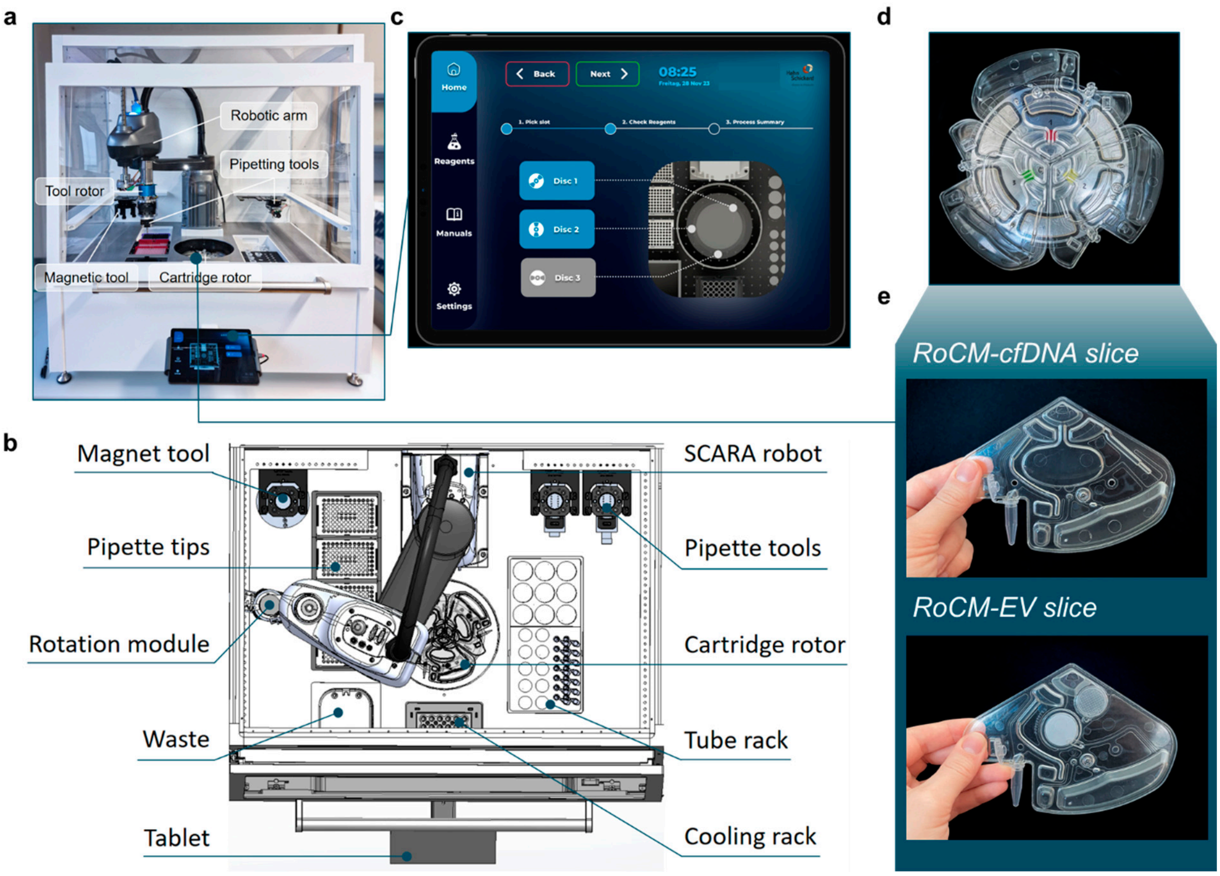

Figure S1: *Technical implementation of RoCM concept. a, Photograph of the RoCM platform. The realised benchtop design of the* RoCM *platform (920 × 800 × 960 mm, W × D × H; approx. 110 kg) integrates all essential components of automated liquid biopsy extraction protocols. b, Top view inside of the RoCM platform with components. The system includes four pipette tip racks, accommodating two times 96 tips, each for 50 µL and 1 mL volumes, a cooling rack capable of controlling temperatures down to 4 °C, and a rotor operating at up to 60 Hz with accelerations of up to 24 Hz/s. Integrated Peltier elements inside the rotor provide on-cartridge temperature control up to 120 °C. The reagent compartment includes tube racks for 10 × 5 mL tubes, 14 × 2 mL tubes, and 9 × 50 mL bottles. c, Tablet screenshot of the graphic user interface. RoCM can operate in a preprogramed mode, which can alternatively be controlled* via *a graphic user interface on a tablet d, Photograph of three assembled RoCM-cfDNA slices on the cartridge rotor. e, Photographs of the RoCM-cfDNA and RoCM-EV slice.*

***Table S1: Decision matrix for concept with rotating SCARA** with pipette with weights ω, total weights ω_{tot}, points P and weighted points P_{ω}.*

**Criteria**	w_{tot} (%)	**Sub-criteria**	w (%)	**rot. SCARA with pipette, floor-mount**		
				**Comment**	**P**	P_w
Performance	30%		30%	Necessary vel and acc values are reached by SCARA. Less leverage bc. of floor-mount.	7	2.1
User-friendliness	25%	Safety/Longevity	5%	Strong wear on system due to dynamic rotation.	5	0.25
		Easy set-up	5%	Plug and play of robot with teach pendant.	8	0.4
		Easy programming	5%	Synchronization scheme has to be developed.	5	0.25
		Level of automation	10%	Automatic pipetting of samples and reagents	10	1
Novelty	15%		15%	Novel concept.	10	1.5
Sustainability	15%	Extensibility	5%	Additional signal lines and end-effector already present.	9	0.45
		Workspace-to-Footprint Ratio	5%	(0.78/0.058) m^2	9	0.45
		Weight	5%	25 kg w/o control unit.	5	0.25
Avoidance of cross-contamination	15%		15%	Disposable tips.	10	1.5
SUM	100%		100%			6.4

Table S2: Decision matrix for concept with rotating Cartesian robot *with pipette with weights ω, total weights ω_{tot}, points P and weighted points P_ω.*

**Criteria**	w_{tot} (%)	**Sub-criteria**	w (%)	**rot. Cartesian robot with pipette, ceiling-mount**		
				**Comment**	**P**	P_w
Performance	30%		30%	Necessary vel and acc values reached. Slightly less dynamics than for SCARA.	8	2.4
User-friendliness	25%	Safety/Longevity	5%	Slightly less wear on system than SCARA	6	0.3
		Easy set-up	5%	Mounting on ceiling necessary. Cable management.	5	0.25
		Easy programming	5%	Synchronization scheme has to be developed, but simple coordinate system.	7	0.35
		Level of automation	10%	Automatic pipetting of samples and reagents	10	1
Novelty	15%		15%	Novel concept.	10	1.5
Sustainability	15%	Extensibility	5%	Additional signal lines are possible.	8	0.4
		Workspace-to-Footprint Ratio	5%	(5805 mm axes travel/6000 mm axes length)	3	0.15
		Weight	5%	24 kg w/o control unit.	5	0.25
Avoidance of cross-contamination	15%		15%	Disposable tips.	10	1.5
SUM	100%		100%			6.35

Channel dimensions of cartridge designs

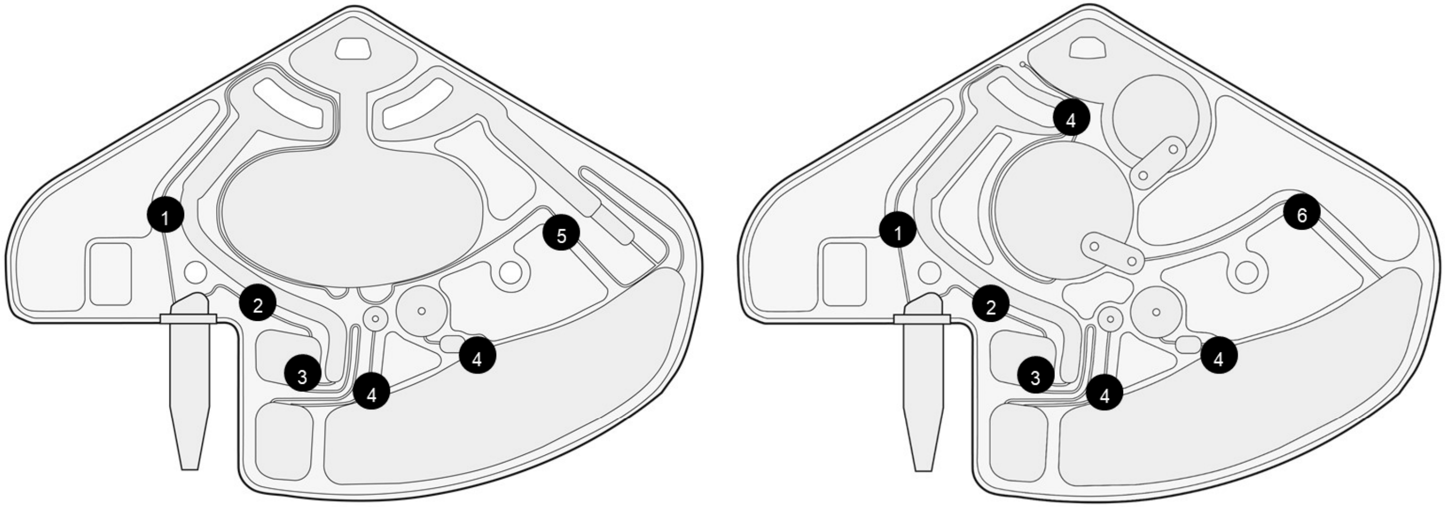

#	Description	Dimensions w x h [µm]
**1**	Extract transfer channel	230 x 200
**2**	Underpressure chamber to tube connection channel	400 x 250
**3**	Underpressure chamber to inlet connection channel	230 x 200
**4**	Vent channel	300 x 300
**5**	RoCM-cfDNA waste transfer channel	440 x 400
**6**	RoCM-EV waste transfer channel	430 x 400

Figure S2: Channel dimensions of cartridge designs. *w: width ; h: height.*

Milled PMMA Cartridge for Volumetric Pipetting Precision Testing

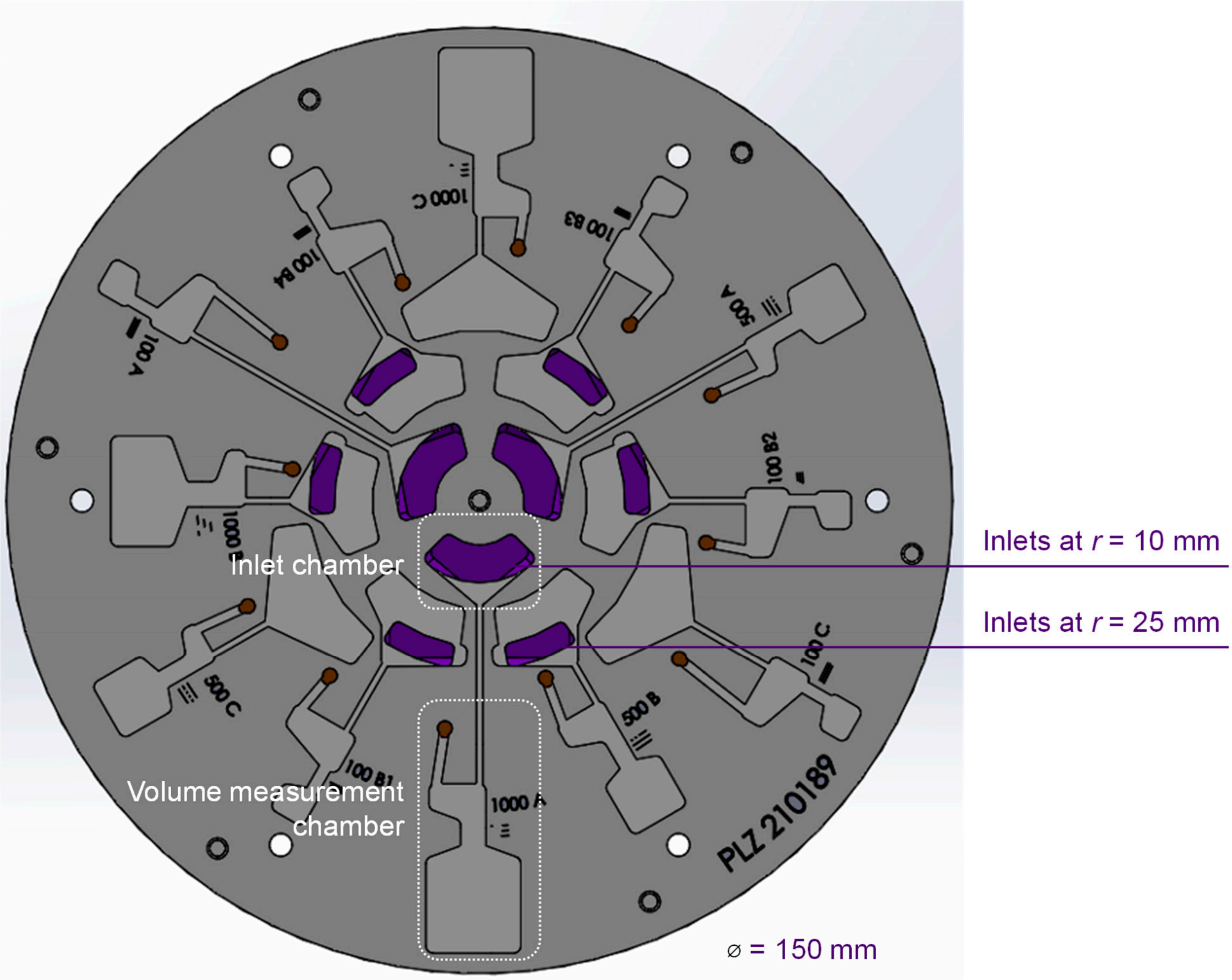

Figure S3: *Milled PMMA cartridge for volumetric pipetting tests at 100 µL, 500 µL, and 1000 µL, conducted at radii of 10 mm and 25 mm. Disks were sealed with pressure-sensitive foil (9795R, 3M), and openings in purple were cut out as inlets. Volumetric measurements were obtained by overlaying stroboscopic images acquired during rotation with the corresponding CAD model, allowing volume estimation based on the observed fluid fill level.*

Binding and magnetic mixing step is most crucial for extraction efficiency

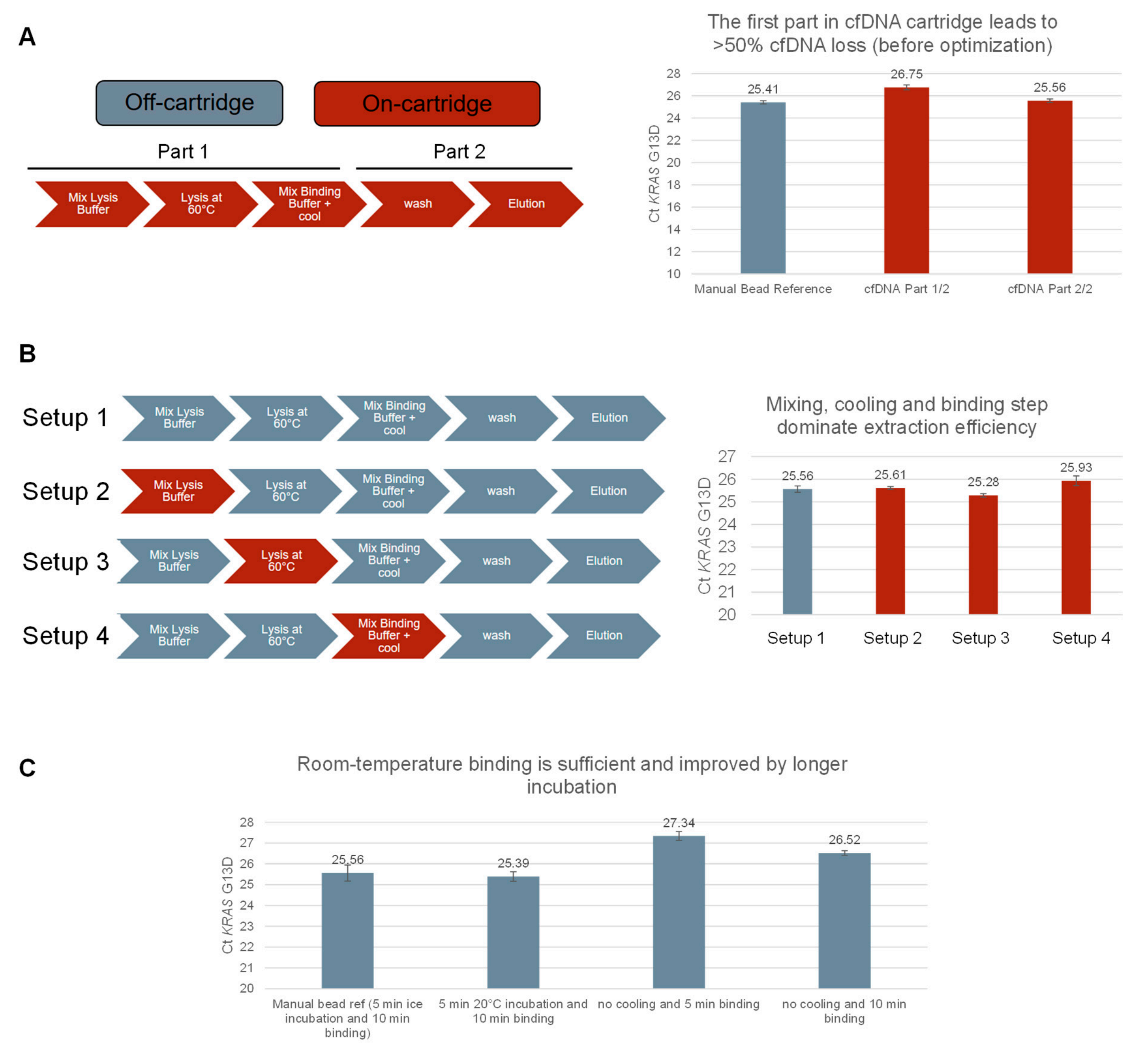

Figure S4: A, *To evaluate and optimize the cfDNA extraction protocol, the cartridge workflow was divided into two parts. Each part was independently tested both on-cartridge and off-cartridge. ctDNA$_{surrogate}$ was spiked into 1 mL of healthy donor plasma and analyzed by qPCR in technical triplicates. Comparison of cycle threshold (Ct) values served as a proxy for extraction efficiency. Notably, when Part 1 of the protocol was executed on-cartridge, a substantial reduction in extraction efficiency was observed.* ***B,*** *To localize the source of extraction inefficiency, Part 1 was further subdivided into three discrete steps (Setup 2 to Setup 4), each of which was individually tested on-cartridge and compared to manual reference (Setup 1). These experiments identified the final step of Part 1 (cold incubation on ice followed by incubation of magnetic beads with binding buffer), as the critical determinant.* ***C,*** *Further dissection of this step revealed that the ice incubation could be replaced with incubation at 20 °C, whereas the bead-binding process itself was found to be the principal contributor to reduced efficiency (e.g. binding time).*

Valve actuation – concept study of robotic waste transfer valve actuation for repeated liquid holding and transfer.

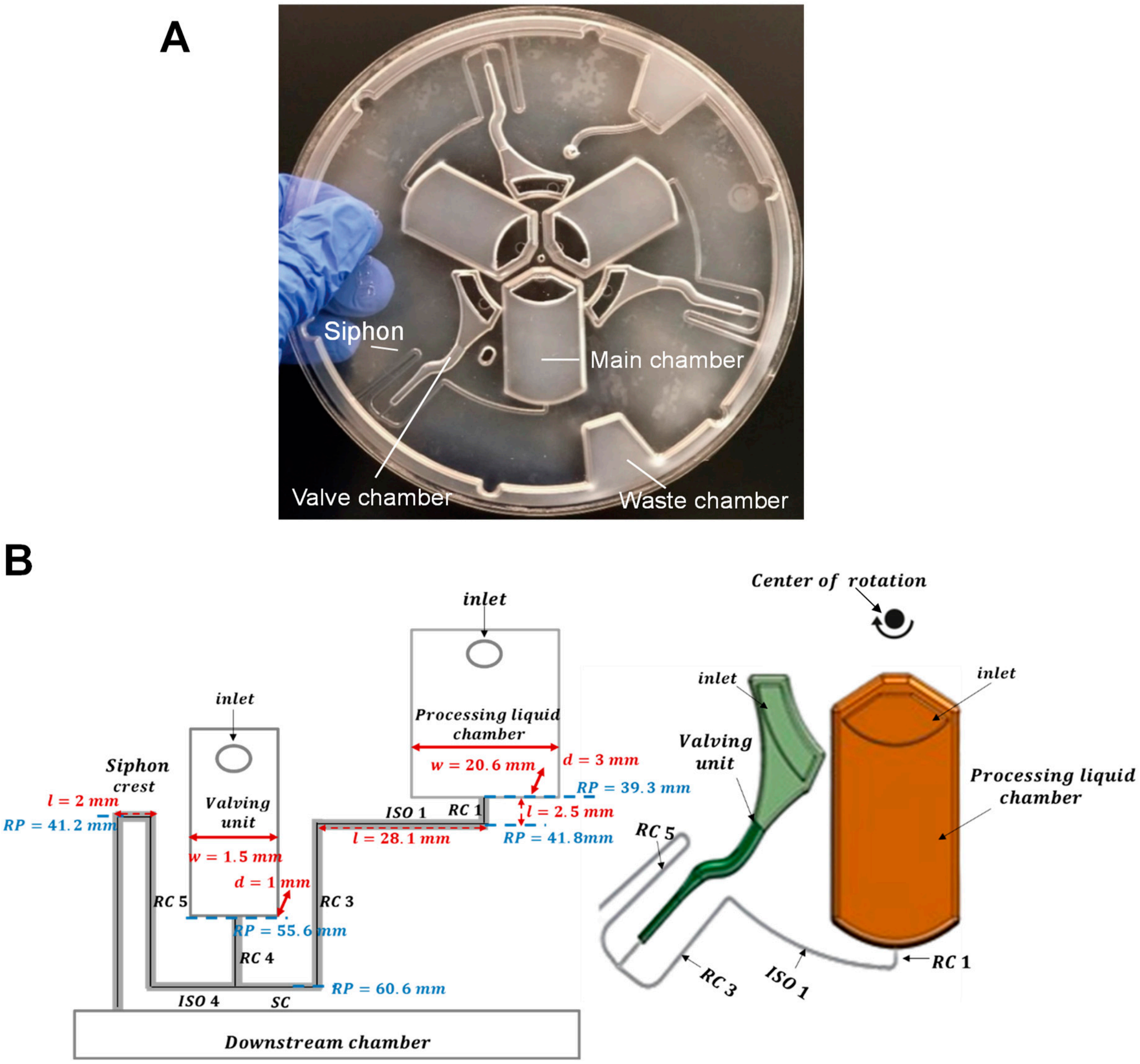

Figure S5: A, COC foil cartridge for evaluation of robotic valve actuation for repeated liquid holding and transfer. B, Design schematic (left) and CAD design model (right). RC: radial channel, ISO: isoradial channel, SC: straight channel, d: chamber depth, w: chamber width.

Table S3: *RoCM-cfDNA slice extraction protocol with used frequencies, temperatures and robotic operations. RT: room temperature*

Step	Description	Loop	Argument [Hz x 100; acc. Hz/s x 100; decc. Hz/s x 100]	Robotic operation at 5 Hz
**1**	Pinning of valves		0; 500; 500	20 µL H_2O to waste transfer valves
**2**				120 µL to extract transfer valves
**3**			4000; 1000; 1000 & Wait 5s	
**4**	Loading of main chamber		500; 500; 500	100 µL Proteinase K to main chamber
**5**				1000 µL plasma to main chamber
**6**	Proteinase-mixing	3x	2000; 2000; 2000 & Wait 1s	
**7**			200; 2000; 2000 & Wait 6s	
**8**	Loading of main chamber		500; 500; 500	800 µL buffer ACL to main chamber
**9**	Thorough mixing	20x	2000; 2400; 2400 & Wait 1s	
**10**			200; 2400; 2400 & Wait 6s	
**11**	Temp on		65 °C	
**12**	Mixing for lysis	30 min	2000; 2000; 2000 & Wait 1s	
**13**			500; 2000; 2000 & Wait 6s	
**14**	Temp off		StopTempControl	
**15**	Loading of main chamber		500; 500; 500	40 µL beads to main chamber
**16**				1800 µL buffer ACB to main chamber
**17**	Thorough mixing	15x	2000; 2000; 2000 & Wait 1s	
**18**			300; 2000; 2000 & Wait 6s	
**19**			300; 500; 500	
**20**				Magnet on (non-rotating)
**21**	Temp on		20 °C	

Step	Description	Loop	Argument [Hz x 100; acc. Hz/s x 100; decc. Hz/s x 100]	Robotic operation at 5 Hz
**22**	RT equilibration	5x	Wait 5 min at 10 Hz	
**23**		10 min	300; 2000; 2000 & Wait 6s	
**24**			2000; 2000; 2000 & Wait 1s	
**25**				Magnet off
**26**	Temp off		StopTempControl	
**27**	Bead sedimentation		4000; 1500; 1500 & Wait 40s	
**28**	Waste transfer 1		500; 500; 500	140 µL H2O to waste transfer valves
**29**			4000; 500; 200	
**30**	Re-pinning of waste transfer valve		500; 500; 500	10 µL H2O to waste transfer valves
**31**			4000; 1000; 1000 & Wait 5s	
**32**	Loading of main chamber		500; 500; 500	600 µL Buffer ACW1 to Main
**33**				Magnet on (non-rotating)
**34**	Flush down to waste chamber		2000; 500; 500 & Wait 5 s	
**35**	Bead resuspension & wash	2x	1500; 1500; 1500 & Wait 3s	
**36**			300; 1500; 1500 & Wait 6s	
**37**				Magnet off
**38**	Bead sedimentation		4000; 1500; 1500 & Wait 40s	
**39**	Waste transfer 2		500; 500; 500	140 µL H2O to waste transfer valves
**40**			4000; 500; 500 & Wait 10s	
**41**	Repinning of waste transfer valve		500; 500; 500	10 µL H2O to waste transfer valves
**42**			4000; 1000; 1000 & Wait 5s	
**43**	Loading of main		500; 500; 500	750 µL buffer ACW2 to main chamber

Step	Description	Loop	Argument [Hz x 100; acc. Hz/s x 100; decc. Hz/s x 100]	Robotic operation at 5 Hz
**44**				Magnet on (non-rotating)
**45**	Flush down to waste chamber		2000; 500; 500 & Wait 5 s	
**46**	Bead resuspension & wash	2x	1500; 1500; 1500 & Wait 3s	
**47**			300; 1500; 1500 & Wait 6s	
**48**				Magnet off
**49**	Bead sedimentation		4000; 1500; 1500 & Wait 40s	
**50**	Waste transfer 3		500; 500; 500	140 µL H_2O to waste transfer valves
**51**			4000; 500; 500 & Wait 10s	
**52**	Re-pinning of waste transfer valve		500; 500; 500	10 µL H_2O to waste transfer valves
**53**			4000; 1000; 1000 & Wait 5s	
**54**	Loading of Main		500; 500; 500	750 µL EtOH to main chamber
**55**				Magnet on (non-rotating)
**56**	Flush down to waste chamber		2000; 500; 500 & Wait 5 s	
**57**	Bead resuspension & Wash	2x	1500; 1500; 1500 & Wait 3s	
**58**			300; 1500; 1500 & Wait 6s	
**59**				Magnet off
**60**	Bead sedimentation		4000; 1500; 1500 & Wait 40s	
**61**	Waste transfer 4		500; 500; 500	140 µL H_2O to waste transfer valves
**62**			4000; 500; 500 & Wait 10s	
**63**	Repinning of waste transfer valve		500; 500; 500	10 µL H_2O to waste transfer valves
**64**			4000; 1000; 1000 & Wait 5s	
**65**	Temp on		56 °C	
**66**	Evaporation of EtOH	10x	500; 500; 500 & Wait 60s	

Step	Description	Loop	Argument [Hz x 100; acc. Hz/s x 100; decc. Hz/s x 100]	Robotic operation at 5 Hz
**67**	Temp off		StopTempControl	
**68**	Loading of main chamber		500; 500; 500	100 µL Elution buffer to main chamber
**69**	Flush down to waste chamber		2000; 500; 500 & Wait 5s	
**70**				Magnet on (non-rotating)
**71**	Elution	15x	1500; 1500; 1500 & Wait 1s	
**72**			300; 1500; 1500 & Wait 6s	
**73**				Magnet off
**74**	Bead sedimentation		4000; 1500; 1500 & Wait 40s	
**75**	Eluate transfer		500; 500; 500	200 µL H2O to extract transfer valves
**76**			3500; 1000; 2000 & Wait 15s	
**77**			300; 1000; 2000 & Wait 30s	
**78**	Tube collection		2000; 1000;1000 & Wait 50s	

Table S4: *Reaction mixes and PCR conditions for both PCR platforms, including all assays for wild-type (WT) and mutation detection.*

Systems (device with cartridge)	*qPCR (Rotor-Gene Q)*	*dPCR (QIAcuity-Nanoplate 26K)*
Total volume (eluate volume)	10 µL (1 µL)	40 µL (5 µL)
Replicates per samples	3	2
Mastermix	PerfeCTa Multiplex qPCR ToughMix (5x)	QIAcuity High Multiplex Probe PCR Master Mix (4x)
Targets	*KRAS* WT, G13D	*BRAF* WT, V600E, V600K and *NRAS* WT, Q61K, Q61L, Q61R
Primer concentrations	300 nM each	2000 nM for BRAF each, *NRAS* reverse and 1000 nM for *NRAS* forward
Mediator probe concentrations	200 nM each	600 nM each
Universal reporter concentrations	100 nM each	200 nM each
PCR conditions		
Initial denaturation	96 °C for 5 min	96 °C for 5 min
Amplification	40 cylces of 95 °C for 15s and 56 °C for 40s	60 cylces of 95 °C for 15s and 56 °C for 60s

Table S5: *RoCM-EV slice filtration protocol with used frequencies and robotic operations. CEX: cation-exchange-chromatography*

Step	Description	Loop	Argument [Hz x 100; acc. Hz/s x 100; decc. Hz/s x 100]	Robotic operation at 5 Hz
**1**	Pinning of valves		0; 500; 500	120 µL to extract transfer valves
**2**			4000; 1000; 1000 & Wait 5s	
**3**	Loading of main chamber		500; 500; 500	1000 µL diluted CEX-resin-plasma-mix to main chamber
**4**	Filtration		3500; 2000; 2000 & Wait 10 min	
**5**	Shake-mode every 5 min	10x	2000; 2400; 2400	
**6**			500; 2400; 2400	
**7**	Loading of main chamber		500; 500; 500	1000 µL diluted CEX-resin-plasma-mix to main chamber
**8**	Filtration		3500; 2000; 2000 & Wait 15 min	
**9**	Shake-mode every 5 min	10x	2000; 2400; 2400	
**10**			500; 2400; 2400	
**11**	Loading of main chamber		500; 500; 500	500 µL PBS with 5% surface blocking solution to main chamber
**12**	Filtration/washing		3500; 2000; 2000 & Wait 20 min	
**13**	Shake-mode every 5 min	10x	2000; 2400; 2400	
**14**			500; 2400; 2400	
**15**	Loading of main chamber		500; 500; 500	500 µL PBS with 5% surface blocking solution to main chamber
**16**	Filtration/washing		3500; 2000; 2000 & Wait 20 min	
**17**	Shake-mode every 5 min	10x	2000; 2400; 2400	
**18**			500; 2400; 2400	
**19**	Retentate transfer		500; 500; 500	200 µL H2O to extract transfer valves

Step	Description	Loop	Argument [Hz x 100; acc. Hz/s x 100; decc. Hz/s x 100]	Robotic operation at 5 Hz
**20**			3500; 1000; 2000 & Wait 15s	
**21**			300; 1000; 2000 & Wait 30s	
**22**	Tube collection		2000; 1000;1000 & Wait 50s	

Preliminary evaluation of RoCM using spiked plasma samples

To evaluate the extraction efficiency of *RoCM-cfDNA slices*, a dilution series (1:1, 1:10, and 1:100) of $ctDNA_{surrogate}$ was spiked into 1 mL of healthy donor plasma. Samples were split and extracted using either *RoCM-cfDNA slices* or an adapted manual bead-based protocol [1]. Ct values of qPCR measurements for both methods revealed highly comparable extraction efficiencies with both methods across all dilutions (**Supplementary Figure S6a**).

Accordingly, EV recovery was evaluated by comparing *RoCM-EV slices* to SEC as a reference method (**Supplementary Figure S6b**). Plasma samples from healthy donors were spiked with fluorescent EV-CD9-GFP particles at total particle counts ranging from 1.0E+10 to 3.9E+11. Results are sorted from left to right by increasing particle number, as measured by nanoparticle tracking analysis (NTA). For 1.9E+10 particles the measured recoveries by *RoCM-EV slice*s were 32.9% and 25.5%, for 5.7E+10 particles it was 39.0% and 32.7%. The observed positive correlation between particle concentration and EV recovery aligns with previous findings [2]. A logarithmic fit curve between the SEC data points indicates a slightly lower recovery rate by *RoCM-EV slices* (average relative recovery of 82.5%) in comparison to SEC.

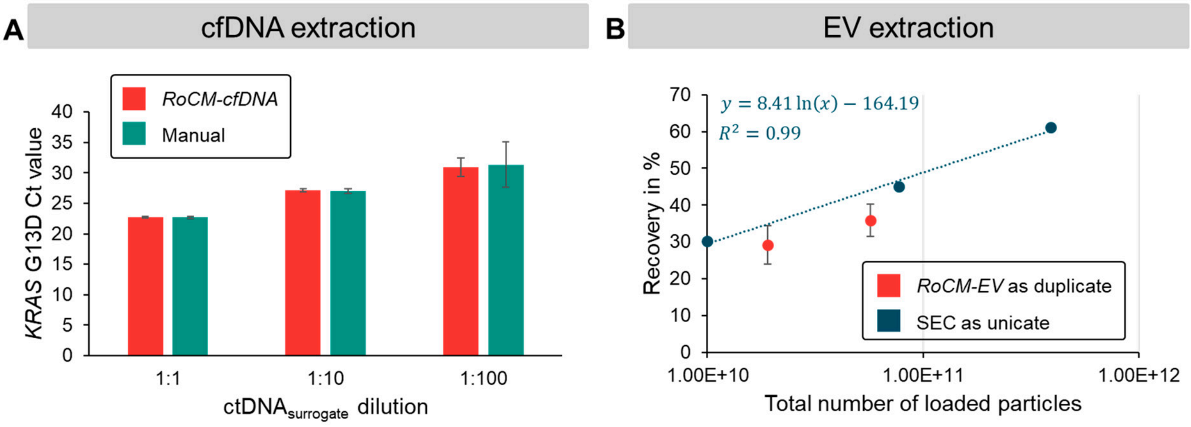

***Figure S6: Preliminary evaluation of RoCM using spiked plasma samples. A**, Comparison of RoCM-cfDNA slices with manual bead-based extraction using a dilution series of $ctDNA_{surrogate}$ in healthy plasma. Extractions were performed in triplicates, and each eluate was measured in qPCR triplicates. **B**, Comparison of RoCM-EV slice eluates with SEC pooled fractions 1 and 2 using spiked EV-CD9-GFP particles. Each sample was measured by NTA as unicates with the established SEC method and as duplicates with the RoCM-EV method. The logarithmic regression line is based on the three SEC values to estimate relative recovery of RoCM-EV slices to SEC.*

Single EV analysis using NanoFCM

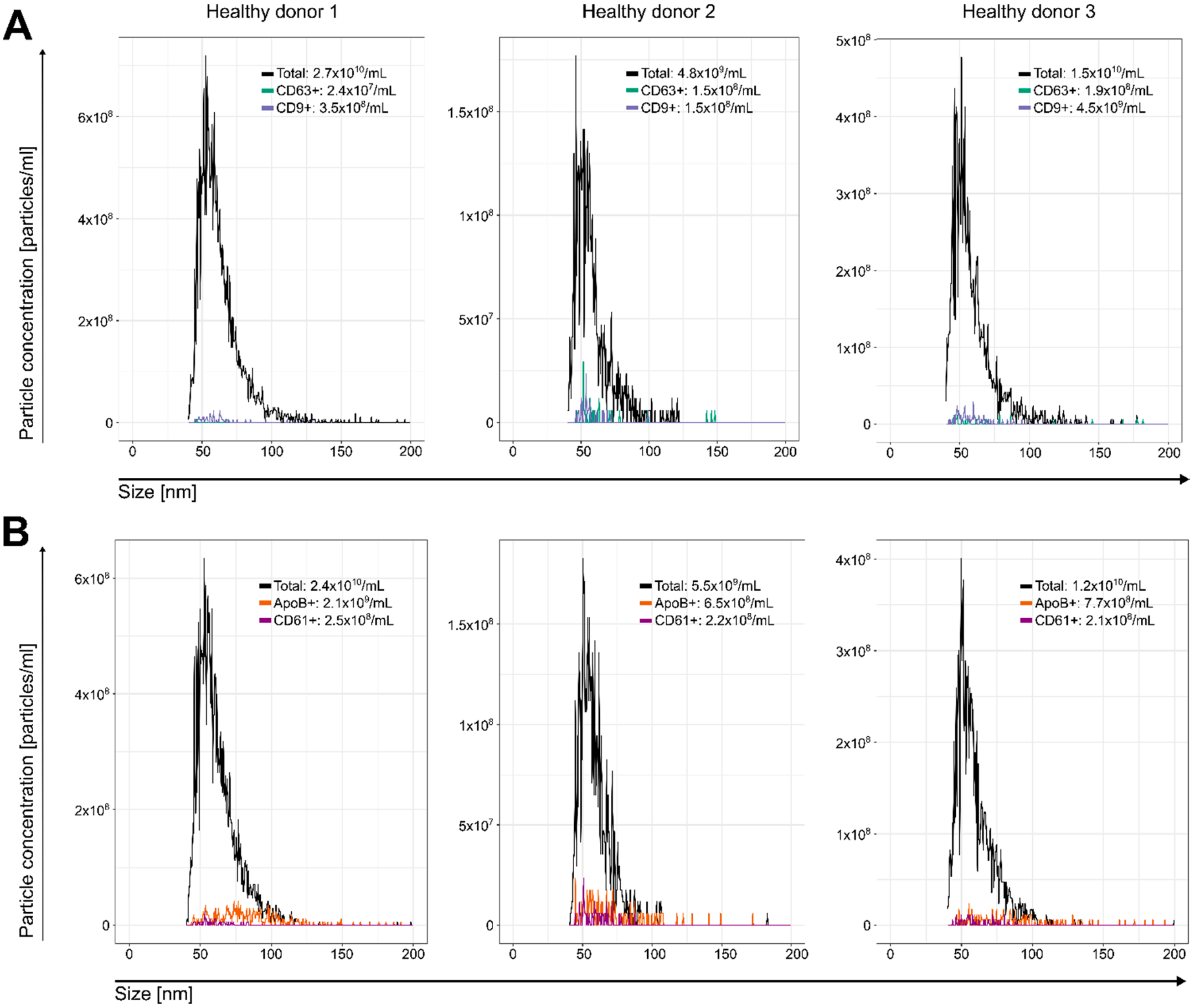

Figure S7: NanoFCM on RoCM-EV retentate: *Representation of the size distribution of EV containing fraction in the retentate obtained from RoCM-EV from three healthy donors. The plots show the total particle number, with each positive subpopulation.* ***A****, Shows CD63, and CD9 positive particles, respectively.* ***B****, Shows CD61, and ApoB positive particles, respectively. Y-axis corresponds to the particle concentration expressed in particles/mL. X-axis corresponds to the size expressed in nm from 40-200 nm.*

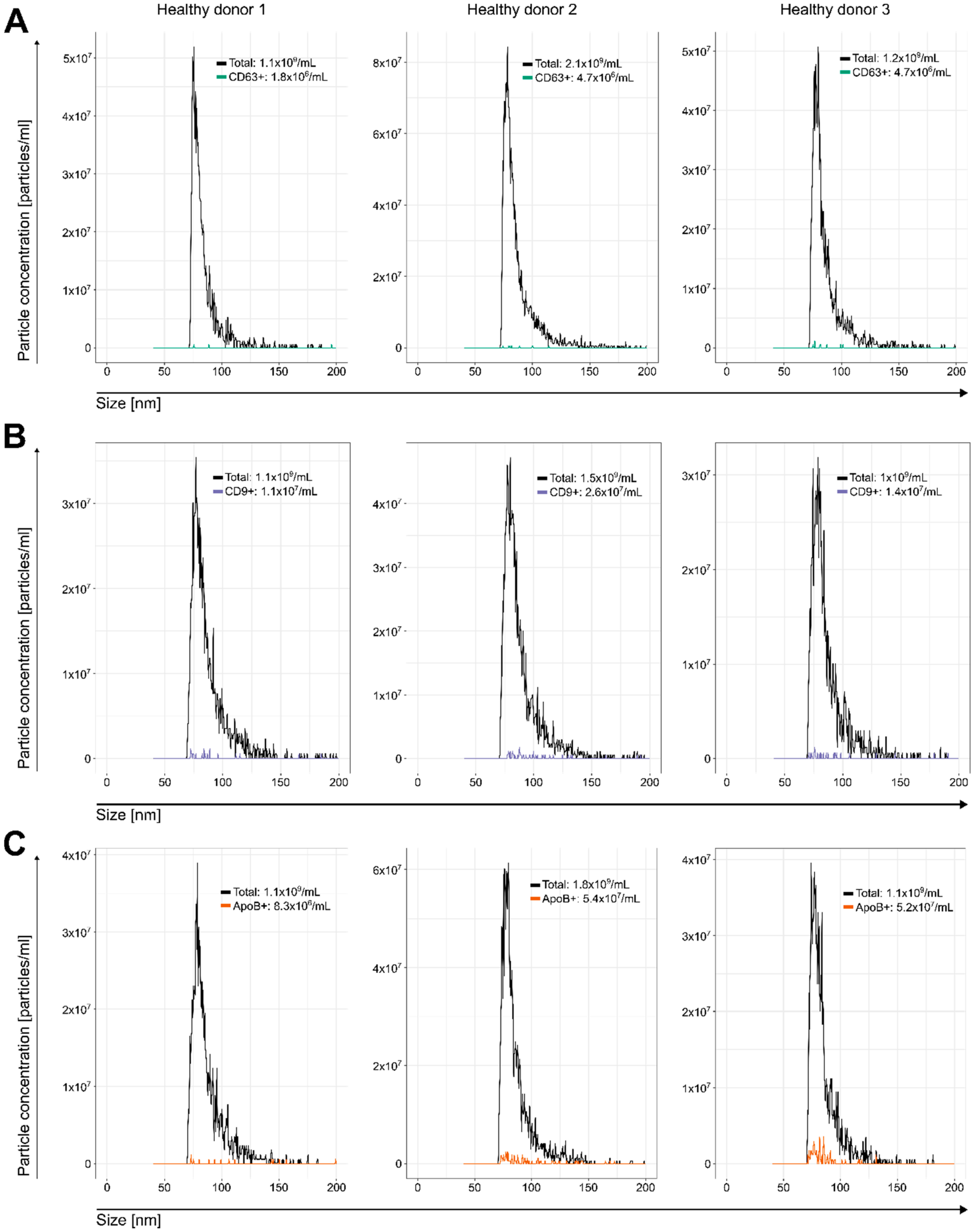

Figure S8: NanoFCM on RoCM-EV filtrate: *Representation of the size distribution of EV containing fraction in the filtrate obtained from RoCM-EV from three healthy donors. The plots show the total particle number, with each positive subpopulation.* ***A,*** *Shows CD63 positive particles.* ***B****, Shows CD9 positive particles.* ***C****, Shows ApoB positive particles. Y- axis corresponds to the particle concentration expressed in particles/mL. X-axis corresponds to the size expressed in nm from 40-200 nm.*

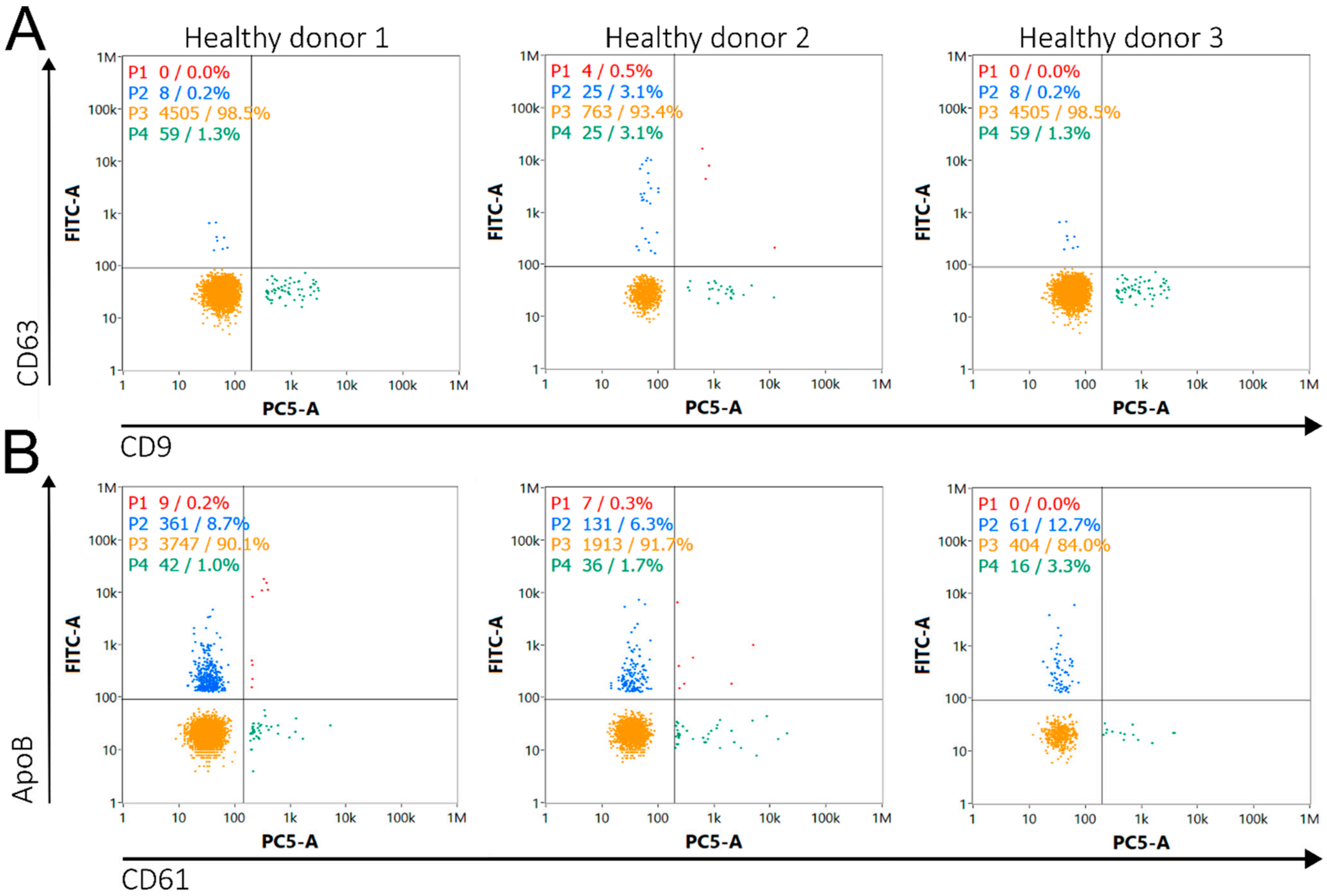

Figure S9: NanoFCM on RoCM-EV retentate: *Representation of the scatter plot of EV containing fraction in the retentate obtained from RoCM-EV from three healthy donors. The plots show the percentage in each positive subpopulation.* ***A****, Shows CD63 positive particles (y-axis on FITC-A) in population 2 (P2), and CD9 positive particles (x-axis on PC5-A) in population 4 (P4).* ***B,*** *Shows CD61 positive particles (x-axis on PC5-A) in P4, and ApoB positive particles (y-axis on FITC-A) in P2. The x-axis corresponds to the size scatter area (SS-A). Both y-axis and x-axis, have a range from 1 to 1,000,000 to record the fluorescent signal. Population 3 (P3) shows in all the plots double negative.*

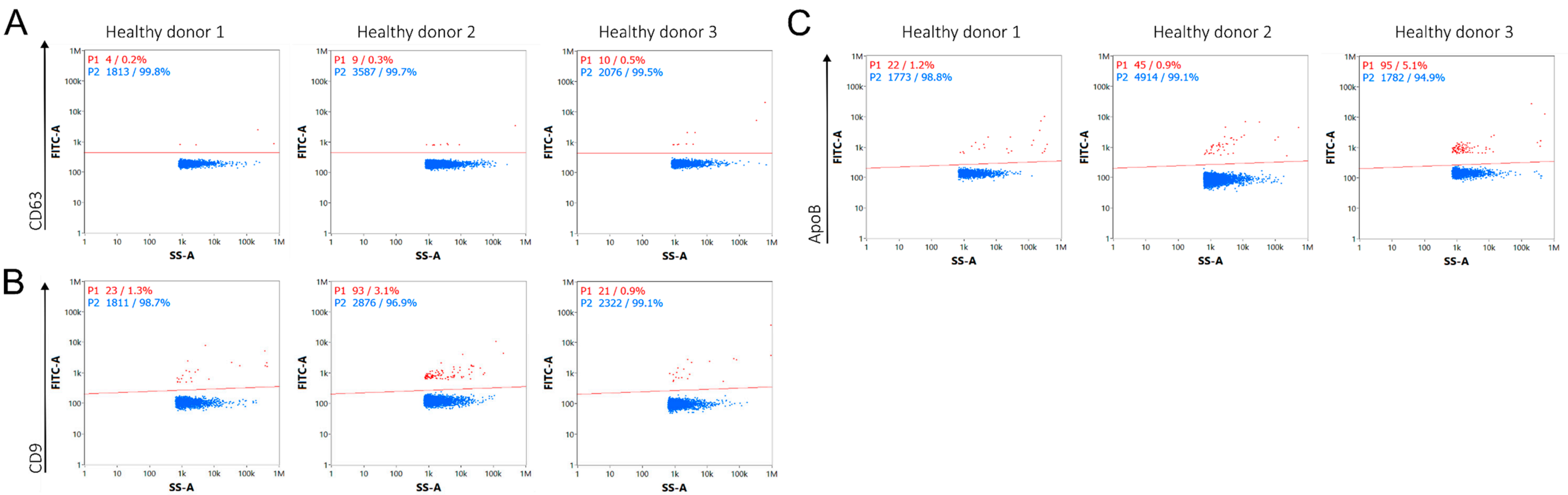

Figure S10: NanoFCM on RoCM-EV filtrate: *Representation of the scatter plot of EV containing fraction in the filtrate obtained from RoCM-EV from three healthy donors. The plots show the percentage in each positive subpopulation. All the plots show in population 1 (P1) the positive particles for the different conditions.* ***A****, Shows CD63 positive particles as P1.* ***B****, Shows CD9 positive particles as P1.* ***C****, Shows ApoB positive particles as P1. The x-axis corresponds to the size scatter area (SS-A). Both y-axis and x-axis, have a range from 1 to 1,000,000 to record the fluorescent signal.*

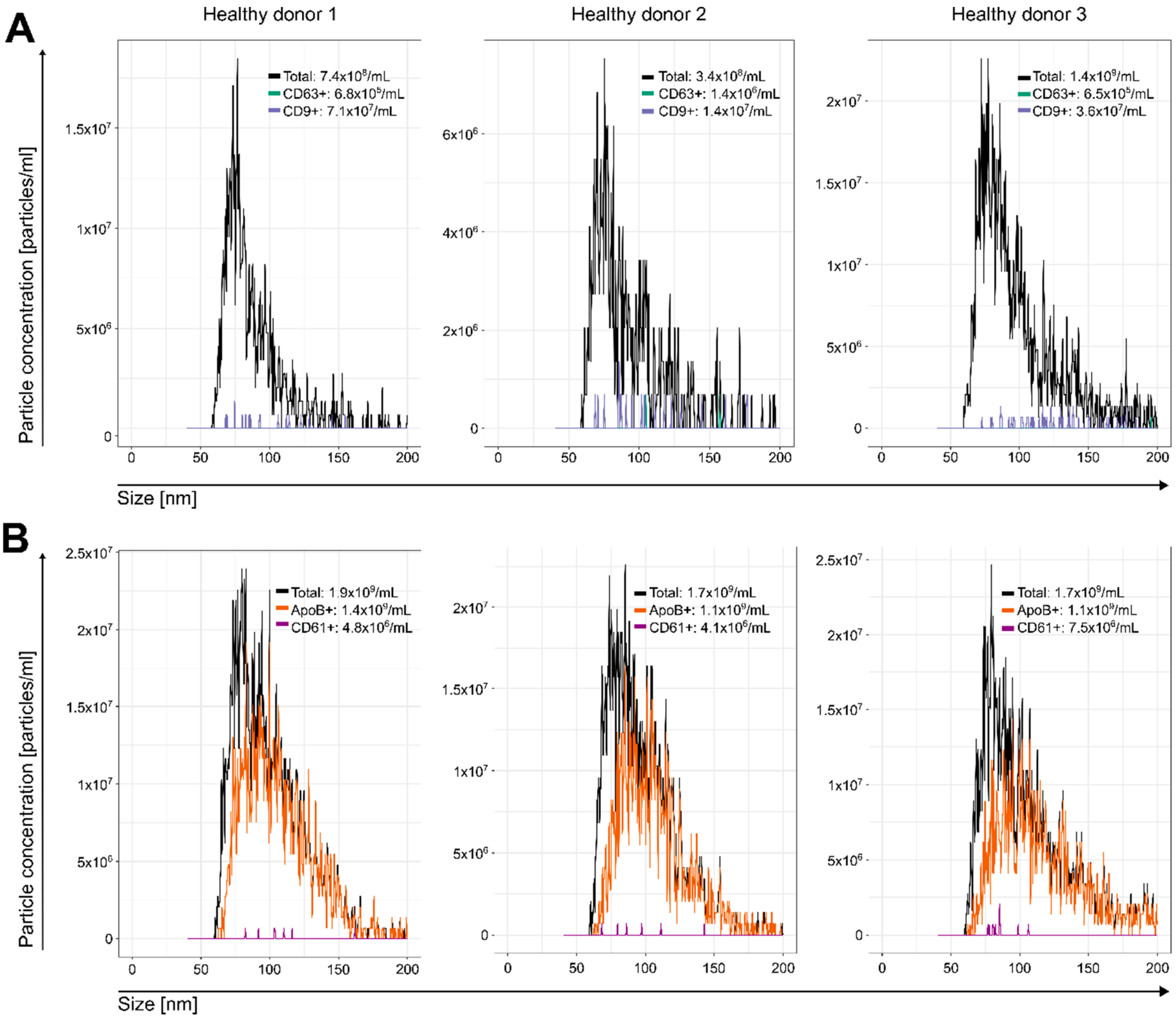

Figure S11: NanoFCM on SEC samples: *Representation of the size distribution of EV containing fraction in the pooled F1-F2 obtained from SEC from three healthy donors. The plots show the total particle number (black), with each positive subpopulation (coloured).* ***A****, Shows CD63, and CD9 positive particles, respectively.* ***B****, Shows CD61, and ApoB positive particles, respectively. Y-axis corresponds to the particle concentration expressed in particles/mL. X-axis corresponds to the size expressed in nm from 40-200 nm.*

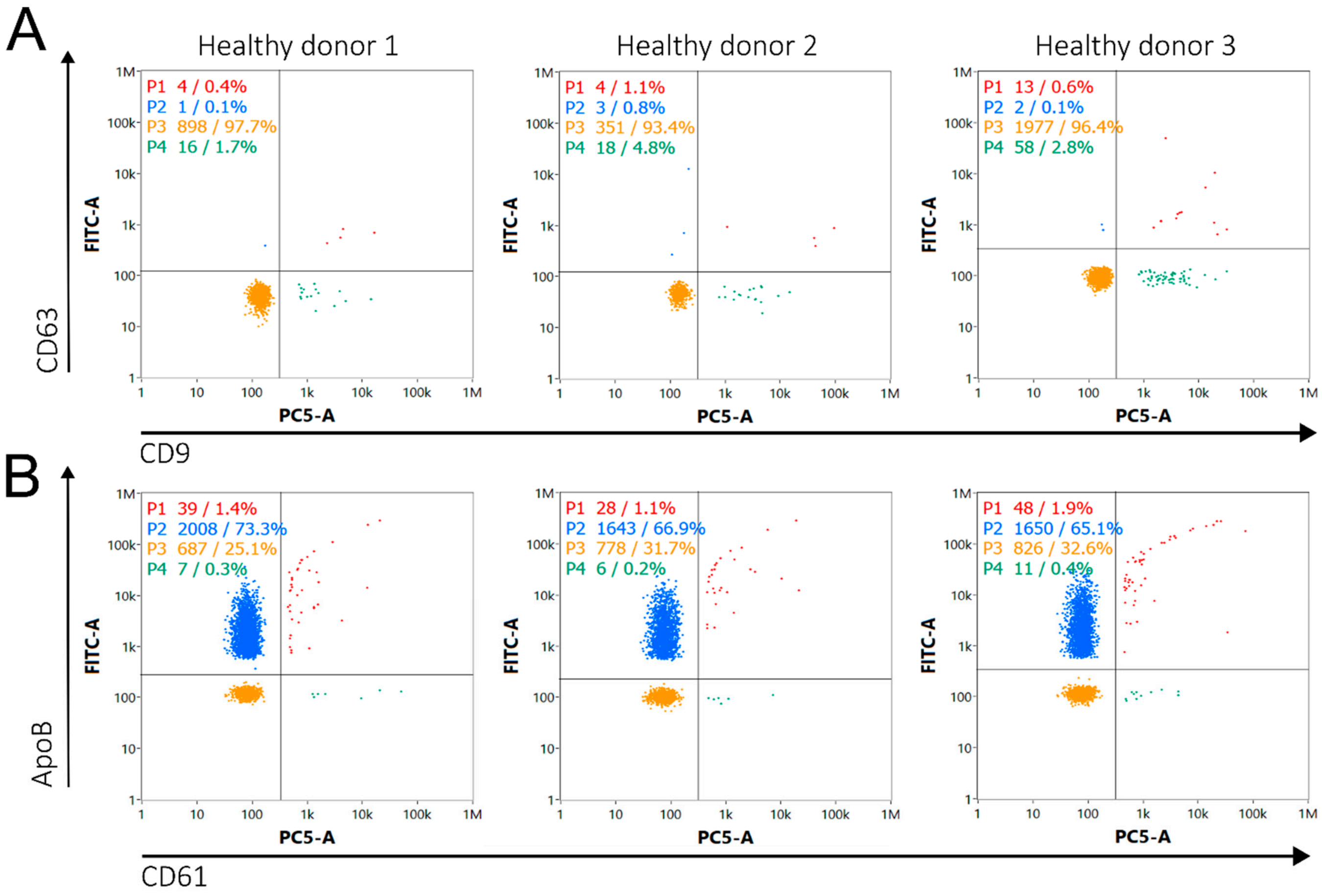

Figure S12: NanoFCM on SEC samples*: Representation of the scatter plot of EV containing fraction in the pooled F1-F2 obtained from SEC from three healthy donors. The plots show the percentage in each positive subpopulation.* ***A****, Shows CD63 positive particles in population 2 (P2), and CD9 positive particles in population 4 (P4). CD63 is y-axis on FITC-A, while CD9 is y-axis on PC5-A.* ***B****, Shows CD61 positive particles in population 4 (P4), and ApoB positive particles in population 2 (P2). ApoB is y-axis on FITC-A, while CD61 is x-axis on PC5-A. Both y-axis and x-axis, have a range from 1 to 1 million to record the fluorescent signal. Population 3 shows in all the plots double negative.*

EV associated signal including from filtrates using patient samples

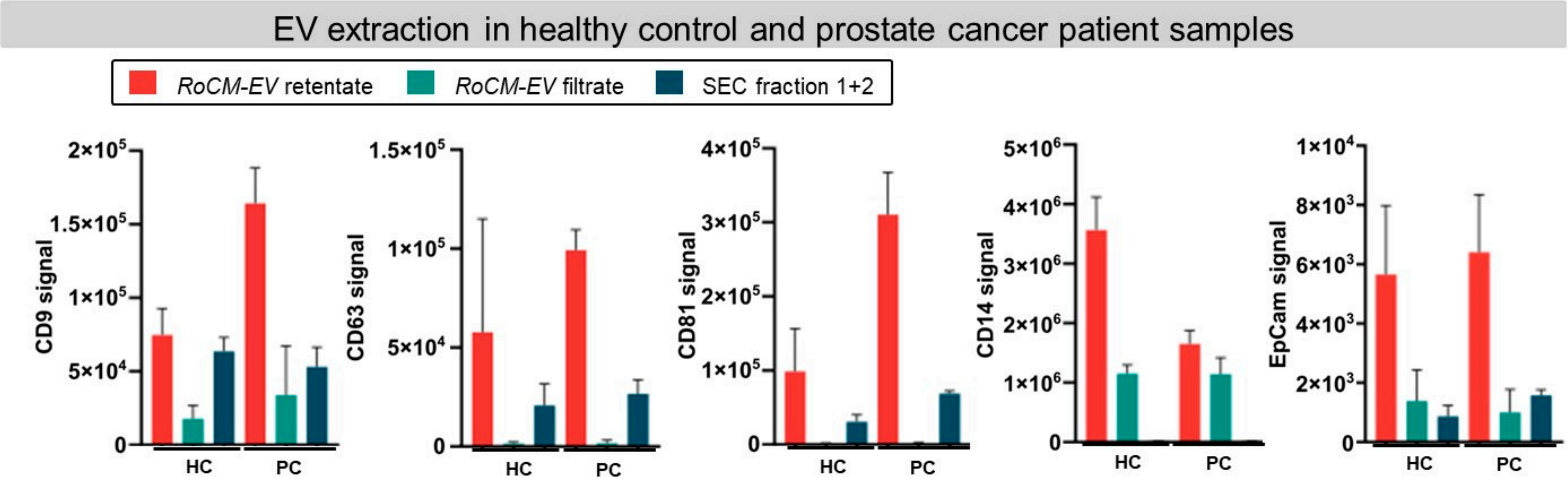

Figure S13: Demonstration of RoCM-EV slices using patient samples: *MSD-based biomarker measurement of healthy controls and prostate cancer patients, each in triplicates. The panel of markers included universal EV biomarkers (CD9, CD63, CD81), the monocyte/macrophage marker CD14, and EpCAM, an epithelial marker commonly overexpressed in cancer. MSD: Meso Scale Discovery; HC: healthy control; PC: Prostate cancer patient.*

Table S6: *Comparison of the three cfDNA extraction methods applied to patient samples at the Department of Hematology and Oncology (University Medical Center Schleswig-Holstein, Lübeck).*

Extraction method	*RoCM*	*EZ2 (QIAGEN)*	*Manual pipetting*
Consumable	*RoCM-cfDNA slice*	*ccfDNA cartridge (QIAGEN)*	*Spin columns (QIAGEN)*
Maximal throughput	*3*	*24*	*24*
Duration	*135 min*	*39 min*	*90-180 min*
Automation	*Yes*	*Yes*	*No*
Analyte-flexibility	*Yes*	*No*	*No*

Table S7: *Comparison of the two EV extraction methods applied to patient samples at the Institute for Infection Prevention and Control (Medical Center, Freiburg).*

Extraction method	*RoCM*	*Fraction collector (IZON)*
Consumable	*RoCM-EV slice*	*qEV original 35 nm gen2 (IZON)*
Maximal throughput	*3*	*10*
Duration	*80 min*	*85 min for one sample + 5 min for other each sample*
Automation	*Yes*	*Semi*
Analyte-flexibility	*Yes*	*No*

References

1. Schlenker, F.; Juelg, P.; Lüddecke, J.; Paust, N.; Zengerle, R.; Hutzenlaub, T. Nanobead handling on a centrifugal microfluidic LabDisk for automated extraction of cell-free circulating DNA with high recovery rates. *Analyst* **2023**, *148*, 932–941, doi:10.1039/D2AN01820A.
2. Nouvel, J.; Bustos-Quevedo, G.; Prinz, T.; Masood, R.; Daaboul, G.; Gainey-Schleicher, T.; Wittel, U.; Chikhladze, S.; Melykuti, B.; Helmstaedter, M.; et al. Separation of small extracellular vesicles (sEV) from human blood by Superose 6 size exclusion chromatography. *J. Extracell. Vesicles* **2024**, *13*, e70008, doi:10.1002/jev2.70008.
3. Arjmand, E.M.; Grether, G.; Bustos-Quevedo, G.; Atanga, J.; Sánchez-Martín, P.; van Deun, J.; Hutzenlaub, T.; Nazarenko, I.; Paust, N.; Lueddecke, J. *VDisk: Microfluidic Cartridge for Multimodal High-Yield, High-Purity Isolation of Extracellular Vesicles from up to 1 mL of Plasma*, 2025.
